# Municipal Solid Waste (MSW)-Compost Amendment Increases Diversity, Functional Activities, and Network Connectivity of a Vineyard Soil Microbiota

**DOI:** 10.3390/microorganisms14061372

**Published:** 2026-06-21

**Authors:** Massimiliano Cardinale, Fabio Minervini, Francesco Maria Calabrese, Margherita Chiarini, Matteo Bernardi, Maria Calasso, Mohammad Yaghoubi Khanghahi, Piergiorgio Romano, Gianni Zorzi, Maria De Angelis, Laura Rustioni

**Affiliations:** 1 Department of Biological and Environmental Sciences and Technologies, University of Salento, 73100 Lecce, Italy; piergiorgio.romano@unisalento.it (P.R.); laura.rustioni@unisalento.it (L.R.); 2 Department of Soil, Plant and Food Sciences, University of Bari “Aldo Moro”, 70126 Bari, Italy; fabio.minervini@uniba.it (F.M.); francesco.calabrese@uniba.it (F.M.C.); margherita.chiarini@uniba.it (M.C.); matteo.bernardi@uniba.it (M.B.); maria.calasso@uniba.it (M.C.); maria.deangelis@uniba.it (M.D.A.); 3 Department of Agricultural, Forestry, Food and Environmental Sciences, University of Basilicata, 85100 Potenza, Italy; mohammad.yaghoubi@unibas.it; 4 Heracle S.r.l., 30020 Eraclea, Italy; gianni.zorzi@yahoo.it

**Keywords:** municipal solid waste (MSW)-compost, vineyard soil management, circular economy, sustainable agriculture, soil microbiota, *Devosia*, functional prediction, Tea Bag Index (TBI)

## Abstract

Sustainable agriculture increasingly relies on organic amendments that integrate circular economy principles. Municipal Solid Waste (MSW)-derived compost (MSW-compost) represents a promising candidate as soil amendment in viticulture, yet its impact on soil microbiota remains poorly investigated. This study assessed the effects of MSW-compost application on the bacterial microbiota of a Mediterranean vineyard soil over a twelve-month period, comparing two application methods (surface mulching and tillage incorporation). Soil DNA was analyzed by 16S rRNA gene metabarcoding, complemented by functional prediction (Picrust2) and the Tea Bag Index to assess soil decomposition activity. MSW-compost significantly increased alpha-diversity and affected beta-diversity (*p* = 0.001) of the microbiota, regardless of the application method, with significant effects persisting throughout the entire observation period despite a clearly diminishing trend. *Devosia* emerged as the hub taxon of the co-occurrence network and was increased by compost addition. MSW-compost application mode remarkably affected the microbial network, with mulched treatment leading to a more complex, denser, and more interconnected network. While a similar number of taxa were increased or decreased, functional prediction revealed a notable enrichment of metabolic pathways, both synthetic and degradative, in the MSW-compost amended samples; this finding was supported by the enhanced red tea decomposition data (*p* = 0.007). Our results indicate that MSW-compost acts as a beneficial soil amendment, simultaneously enhancing microbial diversity and soil decomposition activity. This study provides novel evidence supporting the use of MSW-compost as a sustainable tool for improving soil microbiological quality in productive vineyards.

## 1. Introduction

Sustainable soil management has become a critical aspect of modern agriculture, with implications extending beyond basic agronomic purposes. Resource circularity is a key point to achieve the final goal of the long-term health of agroecosystems [1]. In this framework, municipal solid waste (MSW)-derived compost (MSW-compost) emerged as a useful candidate resource for the sustainable management of vineyard soils [2]. The use of MSW-compost as an amendment represents a practical agricultural application of the circular economy principles, as it transforms the organic fraction of the urban waste—food leftovers, green residues, and other biodegradable materials—into a valuable soil enhancer [2,3,4]. It is important to note that this approach aligns with the European Union directives of Agenda 2030 on waste reduction [5], as it reduces pressure on landfills simultaneously delivering agronomic benefits, such as increasing soil fertility, improving its structure, and supporting soil microbial communities [6,7]. As such, MSW-compost supports the transition toward both a resource-efficient agriculture and a virtuous sustainable soil management practice [8]. When incorporated into the soil by tillage, the compost is expected to positively impact the soil characteristics, such as organic matter content, bulk density, microbial activity, and infiltration rate [4,9]. When used as surface mulch, in addition to similar effects on soil nutrient and microbiological activity [10], organic compost also modulates soil temperature by reducing the solar radiation effects: studies conducted in Mediterranean vineyards showed that MSW-compost mulching maintained soil temperatures significantly lower and reduced the amplitude of daily thermal fluctuations in comparison to both bare soil and polyethylene film during summer months, thus reducing heat stress on root systems and thereby preserving root functionality and plant resilience [11,12], which could also be reflected on the microbial component of the soil. Moreover, a range of allelopathic compounds released during organic matter decomposition can inhibit the germination and early growth of weed species, particularly those with small seeds [13,14,15]. This anti-germinative property has been confirmed even when compost was incorporated into the soil rather than applied as surface mulch [16]. 

MSW-compost also plays a significant role in modulating the soil microbiota. For example, its application stimulated the colonization of grapevine roots by native arbuscular mycorrhizal fungi [17], important root symbionts with relevant effects on grapevine fitness and productivity [18]. The assessment of how agronomic practices, such as compost application, shape the soil microbiome has been greatly advanced by the advent of high-throughput sequencing and, in particular, amplicon-based metabarcoding approaches. By targeting informative marker genes—16S rRNA gene for prokaryotes and ITS region for fungi—metabarcoding enables the characterization of complex microbial communities from environmental samples with deep resolution and high throughput, making it possible to reveal treatment-related shifts in community composition, diversity, and structure [19]. This makes metabarcoding particularly well-suited for assessing the responses of soil microbiota to agricultural amendments, including changes in alpha- and beta-diversity indices and relative abundance of taxa associated with different treatments [20,21].

Metabarcoding also makes possible to investigate potential functional and ecological aspects of microbial communities [22]. As a complementation of the taxonomic profiling, functional prediction allows inferring the metabolic potential of microbial communities directly from marker gene data; freely available tools like PICRUSt2 [23], Tax4Fun2 [24], and FUNGuild [25], among others, make use of reference genomes, the KEGG database, and other databases to predict the potential metabolic features at the community level, in terms of enzymes synthesized, functional pathways realized, ecological roles, etc. These represent powerful tools for investigating the possible functional consequences of changes in microbiota composition and have also been applied in vineyard research to assess how different soil management strategies influence ecosystem-level processes [26,27].

Despite the recognized importance of soil microorganisms for soil health and crop productivity [28], still little is known about the effects of MSW-compost on soil microbiota in vineyards. Therefore, the aim of this study was to investigate the effects of MSW-compost application on the soil bacterial microbiota of a Mediterranean vineyard, by integrating 16S rRNA gene metabarcoding with functional prediction, and confirming the results with observations on organic matter decomposition activity measured with the Tea Bag Index (TBI) [29], a standardized method, previously validated in several studies including vineyards [27,30,31], which uses green and red tea to measure the actual soil decomposition activity of labile and recalcitrant compounds, respectively. We also compared the effects of MSW-compost application as mulching or integrated in soil by tillage. We hypothesized that the MSW-compost would modulate the soil microbiota in a positive way by (i) increasing its diversity, (ii) modifying its structure, (iii) improving its functionality, and (iv) strengthening the microbial network connections in an application mode-dependent way.

## 2. Material and Methods

### 2.1. Vineyard, Experimental Design and Sampling Strategy

The field trial was carried out in Salento, an important viticultural area of the Italian region Apulia, in a productive vineyard (Lat: 40°21′09.6″ N—Lon: 17°25′37.3″ E) belonging to the private company Tenute Eméra (Lizzano, Italy). Vines are planted in rows, with a spacing of 0.9 m intra-row and 2.2 m inter-row between the plants (13-year-old *Vitis vinifera* cv. Primitivo vines grafted on 1103 Paulsen rootstocks). The training system is the vertical shoot position, pruned as spur cordon. Further characterizations of the pedoclimatic conditions are available in [16].

The MSW-compost used in this study (Figure S1A) was obtained from the operative district of the company Heracle S.r.l. (Erchie, Italy) and consisted of a composted (static aerated biocells, followed by a maturation phase in biocells) mixture of the organic fraction of municipal solid wastes collected from nearby cities (~88%), green residues (~10%), and organic residues of food processing (~2%). This MSW-compost is commercially available and complies with EU Regulation 2019/1009. The physicochemical parameters of the MSW-compost batch used in this study are indicated in Table S1.

MSW-compost was applied as mulching or tilled on 28 October 2022 (hereafter indicated as “T0”) along one row in the vineyard (intra-row application; Figure S1B), following a randomized block design (Figure S2). Flanking rows, considered as borders, were amended with MSW-compost as well, following a randomized complete block design, but were not analyzed. Moreover, the first and the last block of the rows were also used as borders and left untreated (Figure S2).

After an initial tillage of the entire row, each block consisting of six plants (length = ~6 m; width = ~0.5 m) was either subjected to the amendment with 16 kg plant^−1^ of MSW-compost (corresponding to approximately 80 t ha^−1^ fresh weight), applied intra-row either as mulching or tilled (hereafter “Compost mulch” and “Compost tilled”, respectively), or left unamended (hereafter “No compost”) (Figures S1B and S2). There were three blocks per treatment, for a total of nine randomized blocks.

On the date of the trial establishment (T0), three samples of the yet untreated vineyard soil (hereafter “initial soil” samples) and three samples of the MSW-compost applied (hereafter “compost used” samples) were collected to analyze the initial state. At four successive times (3, 6, 9, and 12 months after MSW-compost application, hereafter indicated also as “T1”, “T2”, “T3”, and “T4”), one soil sample was collected from each block at a 12–15 cm depth, using sterilized instruments, and placed into refrigerated sterile tubes, then transported to the lab and frozen at −20 °C until further processing. In total, 42 soil samples [(4 sampling times × 3 treatments × 3 replicates) + (6 samples initial state)] were collected and analyzed. To collect the soil samples of compost mulch treatment, the mulching layer was temporarily removed to collect the soil sample underneath and then placed back in its position.

### 2.2. Soil DNA Extraction and 16S rRNA Gene Metabarcoding

The DNA was extracted from 500 mg of each soil sample with the “FastDNA SPIN Kit for Soil” and the FastPrep-24TM 5G homogenizer instrument (both MP Biomedicals Inc., Santa Ana, CA, USA), following the manufacturers’ instructions. DNA quantification was obtained with a Qubit™ Flex Fluorometer (Invitrogen Co., Carlsbad, CA, USA). Then, a series of quality and integrity tests were performed using the Agilent 4200 TapeStation System under the Genomic DNA ScreenTape kit (Agilent Technologies, Santa Clara, CA, USA).

Library preparation for 16S rRNA gene sequencing was conducted based on the “16S Metagenomic Sequencing Library Preparation” protocol provided by Illumina Inc. (San Diego, CA, USA). This protocol consists of a series of steps designed for the amplification of the regions of interest, the addition of indexes, and the production of a finished product ready for Illumina MiSeq sequencing setup (Illumina Inc). The V3–V4 region of the 16S gene was amplified with primers containing adapters. The PCR reaction contained 2.5 µL of soil DNA (5 ng/µL), 5 µL of 1 µM forward adapter + primer 341F (5′ TCGTCGGCAGCGTCAGATGTGTATAAGAGACA**GCCTACGGGNGGCWGCAG**, primer sequence in bold), 5 µL of 1 µM reverse adapter + primer 805R (5′ GTCTCGTGGGCTCGGAGATGTGTATAAGAGACAG**GACTACHVGGGTATCTAATCC**, primer sequence in bold), and 12.5 µL of 2× KAPA HiFi HotStart ReadyMix (Hoffmann-La Roche AG, Basel, Switzerland), for a final PCR volume of 25 µL. PCR was performed using the following thermal program: 95 °C initial denaturation for 3 min, followed by 25 cycles at 95 °C (denaturation) for 30 s, 55 °C (annealing) for 30 s, and 72 °C (extension) for 30 s, followed by a final extension at 72 °C for 5 min. PCR products were purified with a magnetic bead purification system using 20 µL Agencourt AMPure XP beads (Beckman Coulter™ Inc., Brea, CA, USA) to eliminate unused primers, nonspecific products, and other residues of the reaction. After the drying step, DNA was eluted with 52.5 µL of 10 mM Tris (pH 8.5) and stored at −20° C until further processing.

For Indexing and Library Purification, 25 µL of 2× KAPA HiFi HotStart ReadyMix (Hoffmann-La Roche AG, Basel, Switzerland) was mixed with 5 µL of Nextera XT Index 1 (N7XX) primer, 5 µL of the Nextera XT Index 2 (S5XX) primer from the Nextera XT Index Kit (Illumina Inc.), 10 µL of PCR-grade water, and 5 µL of purified DNA, for a total reaction volume of 50 µL. Amplification occurred with an initial denaturation at 95 °C for 3 min, followed by 8 cycles of 95 °C for 30 s, 55 °C for 30 s, and 72 °C for 30 s, and a final extension at 72 °C for 5 min. A further magnetic bead-based clean-up (PCR Clean-Up 2) was performed using 30 µL of Agencourt AMPure XP beads (Beckman Coulter™ Inc.), to remove unincorporated indices, free adapters, and undesired small fragments. After magnetic separation, the supernatant was discarded, and the beads were washed twice with 200 µL of freshly prepared 80% ethanol, then air-dried and finally eluted in 25 µL of 10 mM Tris (pH 8.5).

The size and quality of the libraries were assessed using the D1000 assay of the Agilent 4200 TapeStation instrument (Agilent Technologies), by mixing 3 µL of D1000 Sample Buffer with 1 µL of DNA and performing a high-resolution electrophoresis. The libraries of each sample were quantified with the Qubit™ Flex Fluorometer (Invitrogen Co.) and normalized to a final concentration of 4 nM, to ensure equimolar repartition for the sequencing reaction. All libraries were finally pooled together and 20% of a PhiX control library (v3) (Illumina Inc.) was added.

This sample was then loaded into a MiSeq2 cartridge (Illumina Inc., San Diego, CA, USA); following a complete cleaning of both the machine and the flow cell, the sequencing procedure was run on a MiSeq sequencer with 2 × 250 bp paired-end runs (Illumina Inc.).

### 2.3. Bioinformatics Analysis

Following the sequencing procedures, the generated FASTQ files were tested using FastQC to assess their integrity. The sequence data were analyzed with QIIME2, release 2025.10 [32], as follows. Cutadapt version 5.2 [33] was used to both remove the primers and trim any sequence preceding their 5′-end; untrimmed reads were removed. Denoising and chimera removal were performed with Dada2 version 1.26 [34], using the following parameters: --p-trunc-len-f 225 --p-trunc-len-r 215 --p-min-fold-parent-over-abundance 16 --p-max-merge-mismatch 1 --p-min-overlap 10. These parameters were first optimized using a small number of samples, to maximize read retention while maintaining the accuracy and quality of the final sequence dataset. Taxonomical assignment was obtained with the “classify-sklearn” method [35] and the SILVA 138 (Sklearn Version: 1.4.2) database, to create Amplicon Sequence Variants (ASVs). ASVs labeled “mitochondria”, “chloroplast”, “unassigned”, or “Eukaryota”, as well as low-frequency ASVs (with less than 10 reads or occurring in less than 3 samples), were removed. Then, the dataset was rarefied to the smallest sequencing depth [36], corresponding to 10,670 reads: all subsequent analyses (except for the rarefaction curve plotting) were performed using this rarefied dataset.

Taxonomy bar plots at both phylum and genus levels were generated with the software Explicet, version 3.3.31 [37]. All subsequent analyses were performed using QIIME2 taxonomical level 6 (genus-level).

Alpha-diversity metrics (observed taxa, diversity indices, evenness indices, Good’s coverage) and Bray-Curtis dissimilarities were calculated with Explicet, which was also used to plot both Good’s coverage rarefaction curves and a Bray-Curtis dissimilarities heatmap. SPSS version 20 (IBM Corporation, Armonk, NY, USA) was used to perform one-way, two-way, and repeated-measures ANOVA, both followed by Duncan’s post-hoc test (*p* ≤ 0.05), to assess the effect of the treatment on the alpha-diversity indices at each sampling time, separately or along the whole period of investigation, respectively.

The software Statistical Analysis of Metagenomic Profiles (STAMP) version 2.1.3 [38] was used to generate the beta-diversity plots. Differences in community composition among treatments and sampling times, based on Bray-Curtis dissimilarity metrics, were assessed with permutational multivariate analysis of variance—PERMANOVA [39] using the adonis2 function in R version 4.5.3 (R Core Team, 2026; https://www.R-project.org/) with 999 permutations, testing each term after the others (by = ‘margin’) and calculating the significance of treatment, sampling time, and their interaction. Homogeneity of multivariate dispersions was evaluated using the betadisper function [40], with significance assessed by permutation test (999 permutations). To visualize the temporal dynamics of the microbiota composition across treatments, centroid trajectories were computed based on Bray-Curtis dissimilarities. For each treatment and sampling time, the centroid was calculated as the mean position of the three biological replicates. Centroids were then connected with arrows to visualize the directional trajectory of each treatment over time, computing the Euclidean distance to quantitatively assess the convergence over the coordinates of a PCoA plot. These analyses were performed in R using the packages vegan version 2.7-5 [41], phyloseq version 1.55.2 [42], and ggplot2 version 4.0.3 [43], and were restricted to T1–T4 samples, excluding the initial state (T0). Analyses were performed both considering all sampling times together and for each sampling time separately. To account for the longitudinal structure of the experimental design, an additional PERMANOVA was run with permutations restricted within replicates (strata = Replicate).

In order to identify key taxa of the soil microbiota, the genus-level taxa with ≥200 reads in the rarefied dataset, and occurring in at least 12 samples (to ensure robustness of the statistical comparison), were subjected to co-occurrence network analysis [44] using CoNet version 1.1.1, available as an add-on of the software Cytoscape version 3.10 [45]. Compost used samples were excluded from this analysis, to avoid the strong habitat-effect that would likely mask the potential interactions [44]. Pairwise scores were computed for each of the following measures: Bray-Curtis, Kullback-Leibler, Pearson, and Spearman. For each of these measures, 100 permutations (with renormalization and row-shuffling) and 100 bootstrap-resampling scores were generated. Edges with a score outside the 2.5–97.5 percentiles of the bootstraps’ distribution (unstable edges) were removed. The four measure-specific *p*-values were then merged using Brown’s method; variances were pooled by combining the *p*-values. To reduce the risk of accepting casual correlations, only edges with a Benjamini-Hochberg [46] FDR-corrected *p*-value ≤ 0.05 were retained. Moreover, only edges supported by at least three of the four similarity measures were kept as significant correlations, considering that the dissimilarity measures (Bray-Curtis and Kullback-Leibler) are sensitive to outliers and robust to compositionality, while correlation measures (Pearson and Spearman) are biased by compositionality and robust to outliers [44]. The network layout was arranged using the “edge-forced spring embedded” algorithm [47], additionally weighted by *p*-values; this method draws unbiased networks with interconnected nodes close each other and single-linked ones placed apart [48]. Hub nodes, representing taxa with a critical effect in shaping the microbial network [49], were identified based on degree (number of connections), closeness centrality, and betweenness centrality values [49,50]. The topological roles of individual nodes in the co-occurrence network were further evaluated by Zi (measuring how well a node was connected to other nodes in its own module) and Pi (measuring how well a node was connected to nodes in different modules). Based on Zi/Pi scores, nodes were categorized as network hubs (Zi > 2.5; Pi > 0.62), module hubs (Zi > 2.5; Pi < 0.62), connectors (Zi < 2.5; Pi > 0.62), and peripherals (Zi < 2.5; Pi < 0.62), according to [51]. Moreover, co-occurrence networks were also generated per each treatment, in order to assess the effects of MSW-compost amendment on the network connectivity of the soil microbiota.

To assess which genus-level taxa were significantly affected by the MSW-compost addition, No compost samples (all sampling times merged) were compared with compost-treated samples (both Compost mulch and Compost tilled together, all sampling times merged); to ensure robustness of the statistical comparison, low-abundant taxa (less than 200 reads in the rarefied dataset, corresponding to just ~0.05% of the total reads) were removed from this analysis. For this comparison, two approaches were used: (1) the software STAMP, with two-sided Welch’s test, Benjamini-Hochberg FDR correction of the *p*-values (q ≤ 0.05), and a ratio of proportions ≥2 for significance; results were presented as extended error bar plots. (2) ALDEx3 version 1.0.2, as implemented in R, with central log ratio (CLR)-transformation of the data, two-sided Welch’s test, Benjamini-Hochberg FDR correction of the *p*-values (q ≤ 0.05), and a ratio of proportions ≥2 for significance; results were presented as Volcano plots. As an additional confirmation of the results, a LEfSe (Linear Discriminant Analysis Effect Size) analysis, was performed to assess the differential abundance of genus-level taxa between MSW-compost-treated (tilled and mulched samples merged) and untreated samples, averaging all sampling times. LEfSe was performed in R with the package microbiomeMarker version 1.13.2, according to [52].

### 2.4. Functional Prediction

Functional prediction analysis, a method to infer metabolic activities from metabarcoding sequencing data, was performed to assess the effects of MSW-compost addition on the potential metabolic activities of the vineyard soil microbiota. The basic pipeline of Phylogenetic Investigation of Communities by Reconstruction of Unobserved States (Picrust2 version 2.6.3 was used, based on the taxonomical composition of the samples at the genus level, to perform the functional prediction of both KEGG-Orthologs and Functional pathways [23]. Then, the “add_descriptions.py” command was used to add descriptive labels to KEGG-Orthologs and Functional pathways found. According to the Picrust2 description (https://github.com/picrust/picrust2/wiki/How-does-PICRUSt2-work%3F-What-does-it-do%3F, accessed on 4 June 2026), KEGG-Orthologs are molecular functions defined from experimentally characterized genes and proteins in specific organisms, then generalized to other (micro)organisms based on sequence similarity. Next, the functional pathways abundances are calculated as the harmonic mean of the key metabolic pathway reactions (based on KEGG-Orthologs) in each given sample. Therefore, KEGG-Orthologs and functional pathways represent an inference method for assessing the impact of MSW-compost amendment on the vineyard soil functionality based on metabarcoding data and are widely used in microbial ecology studies [23].

The beta-diversity plot based on the Picrust2-inferred functional pathways was created with STAMP. To assess the effect of MSW-compost on both KEGG-Orthologs and Functional pathways inferred by Picrust2, STAMP (Extended error bar plots) and ALDEx3 (Volcano plots) were used, as described above for taxa. To ensure robustness of the statistical comparison, low-abundant KEGG-Orthologs and Functional pathways (number of genes ≤ 0.001% and ≤0.005% of the total Picrust2-predicted number of genes, respectively) were excluded from this analysis.

### 2.5. Tea Bag Index (TBI)

The Tea Bag Index (TBI) [29], a special litterbag method that estimates organic matter decomposition rate, was used to assess the soil microbial decomposition activity. According to the original protocol, Lipton green tea (EAN: 8711327515765) and the Lipton Rooibos (red) tea (EAN: 8711327514348) were used (purchased from Dutchsupermarket, Wageningen, Gelderland, The Netherlands, article codes: THEE049 and THEE056, respectively). Despite some changes in tea bag net manufactures in recent years, the TBI method was proven to be still valid [53]. Green tea contains a higher fraction of easily degradable organic compounds, whereas red tea contains a higher fraction of recalcitrant organic compounds. Tea bags were weighed and then buried at a depth of ~12 cm, one green and one red tea bag per each block of experimental field, in the middle between two vines. After a burial time of 3 months, the tea bags were unburied, dried for 48 h at 65 °C, and then re-weighted, to calculate the weight loss (excluding bag, cord and label). The measure was repeated for four 3-month periods (November–January, February–April, May–July, and August–October). The TBI method has already been successfully used in several vineyard soil studies, such as [11,20,30,31]. Green tea and red tea weight loss were used as a reliable proxy to estimate the occurred degradation of labile organic matter fraction and recalcitrant organic matter fraction, respectively [11,26,27,54]. SPSS was used to perform both one-way and repeated-measures ANOVA, both followed by Duncan’s post-hoc test (*p* ≤ 0.05), to assess the effect of the treatment on the % of tea degradation data, respectively, at each sampling time and along the whole period of investigation.

## 3. Results

### 3.1. Illumina Sequencing

The Illumina sequencing produced, for all the 42 samples, a total of 4,415,028 paired-ends reads. Of these, 4,320,674 reads (~97.86%) passed the primer/trimming step: these primer-cleared sequences were submitted to the European Nucleotide Archive database (www.ebi.ac.uk/ena) under the project accession number PRJEB112833. Continuing with the bioinformatic analysis, 3,566,624 reads (~80.78%) passed the length/quality filtering, 3,201,814 were merged (~72.52%), and 3,162,286 were not chimeric (~71.63%). After the removal of non-bacterial and low-frequency ASVs, 2,863,514 (~64.86%) remained, with a distribution of 139,974 to 10,671 reads per sample. Rarefaction was performed at 10,670 reads per sample. At the genus level (QIIME2 level 6), the Good’s coverage was >99% at the rarefaction point for all samples (Supplementary Figure S3).

### 3.2. Taxonomical Composition

**Figure 1:**
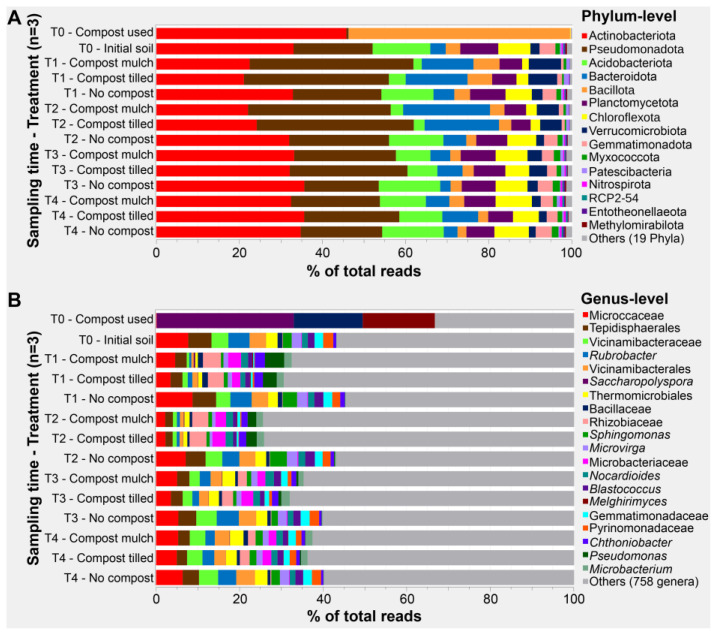
Taxonomical composition of the bacterial microbiota at (**A**) phylum level and (**B**) genus level of compost used, initial vineyard soil, and vineyard soil at 3 (T1), 6 (T2), 9 (T3), and 12 (T4) months after amendment with MSW-compost, mulched or tilled. “No compost” refers to vineyard soil left unamended. Top 15 phyla and top 20 genera are shown, respectively; for genera, the best unequivocal identification obtained by QIIME2-taxonomy was indicated. Each bar represents the average of three replicates (n = 3).

The taxonomical composition showed that the most frequent phylum was Actinobacteriota, followed by Proteobacteriota and Acidobacteriota; however, in the compost samples, the most abundant phylum was Bacillota (Figure 1A). The addition of compost, either tilled or mulch, reduced Actinobacteriota, Acidobacteriota, and Chloroflexota, while increased Pseudomonadota, Bacteroidota, and Verrucomicrobiota (Figure 1A). The most abundant taxa at the genus level were Micrococcaceae, Tepidispherales, Vicinamibacteraceae, and *Rubrobacter*; in the compost samples, the most abundant genus-level taxa, largely dominating the microbiota, were *Saccharopolyspora*, Bacillaceae, and *Melghirimyces* (Figure 1B). No relevant human pathogens occurred in the compost, including *Salmonella*, *Listeria*, *Campylobacter*, *Clostridium*, *Enterococcus*, and *Escherichia*/*Shigella*; all these genera (taxa that can be reasonably detected by the metabarcoding approach) were completely absent in our sequence dataset, with the exception of “*Clostridium sensu strictu*”, which occurred with one single read in the rarefied dataset. A detailed differential abundance analysis of genus-level taxa, as affected by compost amendment, is described in the following paragraphs.

### 3.3. Alpha-Diversity

**Figure 2:**
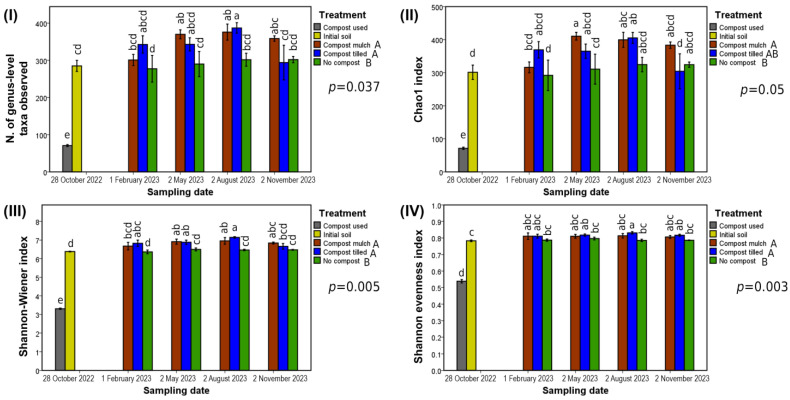
Values of alpha-diversity metrics [(**I**) genus-level taxa observed; (**II**) Chao1 index; (**III**) Shannon-Wiener index; (**IV**) Shannon evenness] found in compost used, initial vineyard soil, and vineyard soil at 3 (1 February 2023), 6 (2 May 2023), 9 (2 August 2023), and 12 (2 November 2023) months after amendment with MSW-compost, either mulched or tilled. “No compost” refers to vineyard soil left unamended. Different low-case letters above the bars indicate significantly different means according to one-way ANOVA followed by Duncan’s post-hoc test (*p* < 0.05). Different capital letters next to the treatment names indicate significantly different means according to repeated-measures ANOVA followed by Duncan’s post-hoc test (*p*-values are shown). Repeated- measures ANOVA was performed on treated samples only (sampling times 3 to 12 months), excluding the initial state (28 October 2022, both initial soil and compost used). Each bar shows the mean ± standard error of three replicates (n = 3).

MSW-compost treatment (both as mulch or tilled) significantly increased the alpha-diversity metrics (observed genus-level taxa, Chao1 index, Shannon-Wiener index, and evenness; Figure 2I−IV, capital post-hoc letters and *p*-value indicated). At single sampling times, overall, significant differences were found from T2 to T4 (Figure 2I−IV), low-case post-hoc letters), whereas the interaction Treatment*Sampling time was not significant (two-way ANOVA, *p* > 0.1 for all metrics). The global effect of compost, i.e., considering tilled and mulch together, was also significant, with even lower *p*-values (N. of genus-level taxa, *p* = 0.009; Chao1, *p* = 0.017; Shannon-Wiener index, *p* = 0.001; Shannon Evenness, *p* = 0.001).

### 3.4. Beta-Diversity

**Figure 3:**
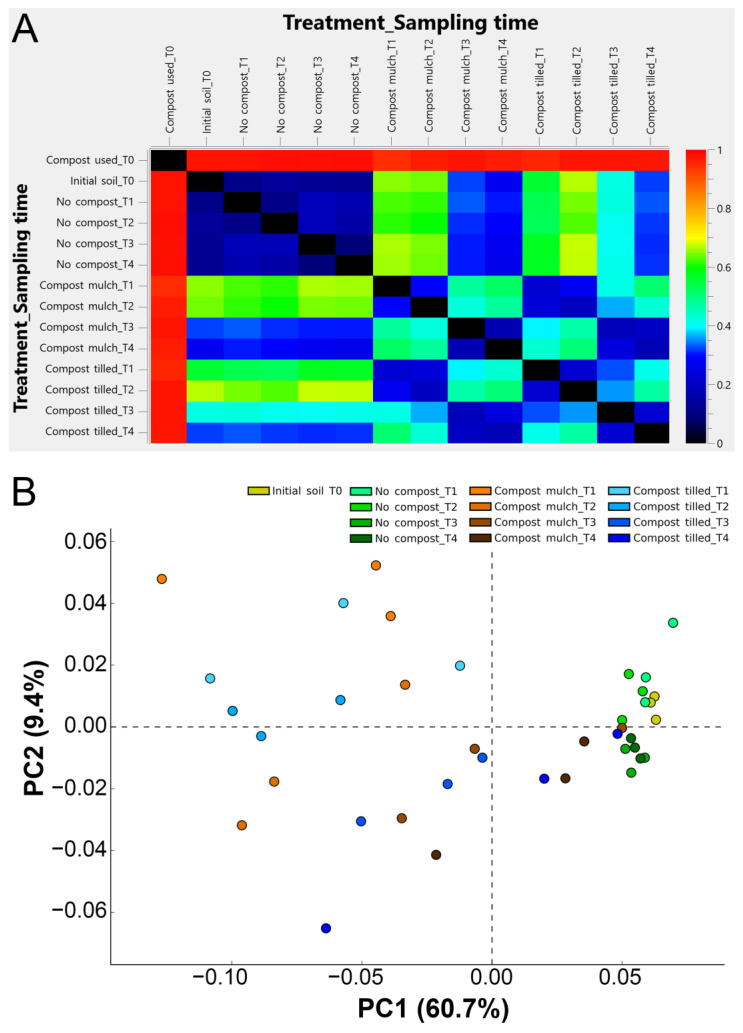
Beta-diversity plots built with sequences of V3–V4 region of the 16S rRNA gene found in the compost used, initial vineyard soil, and vineyard soil at 3 (T1), 6 (T2), 9 (T3), and 12 (T4) months after amendment with MSW-compost, mulched or tilled. “No compost” refers to vineyard soil left unamended. (**A**) Heatmap showing Bray-Curtis dissimilarities between treatments, ordered by treatment and then by sampling time. (**B**) PCA plot generated with STAMP, showing the relative distances between all samples, colored according to the treatment (base colors) and to the sampling times (color tones). In this plot, compost used samples were removed due to their extreme distance, which would flatten all other samples. The PCA plot including compost used samples is shown in Supplementary Figure S4.

MSW-compost treatments (both tilled and mulch) significantly affected the structure of the microbiota (adonis2 *p* = 0.001; Figure 3; Supplementary Figure S4). The dissimilarity between compost-treated samples and No compost samples tended to reduce over time (T1 > T2 > T3 > T4); as clearly evident from the Bray-Curtis (BC) dissimilarity heatmap (Figure 3A), compost-treated samples showed substantial distance from No compost at T1 and T2 (BC dissimilarities = 0.53–0.69), while at T3 and T4 they showed less distance (BC dissimilarities = 0.31–0.44) for both compost mulch and compost tilled (Supplementary Figure S5; Supplementary Table S2). This trend can also be seen in the taxonomical composition, where it is evident that, at T3 and T4, the communities tended to return closer to No compost (Figure 1A,B). As a consequence of this shift over time, compost-treated samples showed a significantly higher beta-dispersion than No compost (*p* = 0.001). However, at each sampling time, adonis2 indicated that compost-treated samples were significantly different from No compost (T1, *p* = 0.013; T2, *p* = 0.021; T3, *p* = 0.030; T4, *p* = 0.020), demonstrating that the effect of compost amendment, as either tilled or mulch, remained until one year after application; indeed, the interaction of Treatment × Sampling time was not significant (*p* > 0.05). When permutations were restricted within replicates, to account for the longitudinal structure of the experimental design, the effect of the treatment remained highly significant (*p* = 0.001), confirming that the observed differences among treatments were robust and not attributable to between-replicate variability.

Compost tilled and compost mulch treatments were not significantly different from each other (*p* > 0.05), indicating that the mode of compost application did not influence the effects on the microbiota. From the beta-diversity plots (Figure 3B), it can be appreciated that all samples from T1 and T2 lie on the upper part of the plot, whereas all T3 and T4 samples are shifted in the lower part (see also temporal trajectories in Supplementary Figure S5): this distribution likely reflects the effect of the sampling time, which was the significant factor driving the sample distribution along the second component (adonis2, *p* = 0.002), with a non-significant beta-dispersion (*p* > 0.05), whereas the interaction Treatment × Sampling time was not significant (adonis2, *p* = 0.20). Any further beta-diversity metrics (Unifrac distances, etc.) produced similar results.

### 3.5. Co-Occurrence Network Analysis

**Figure 4:**
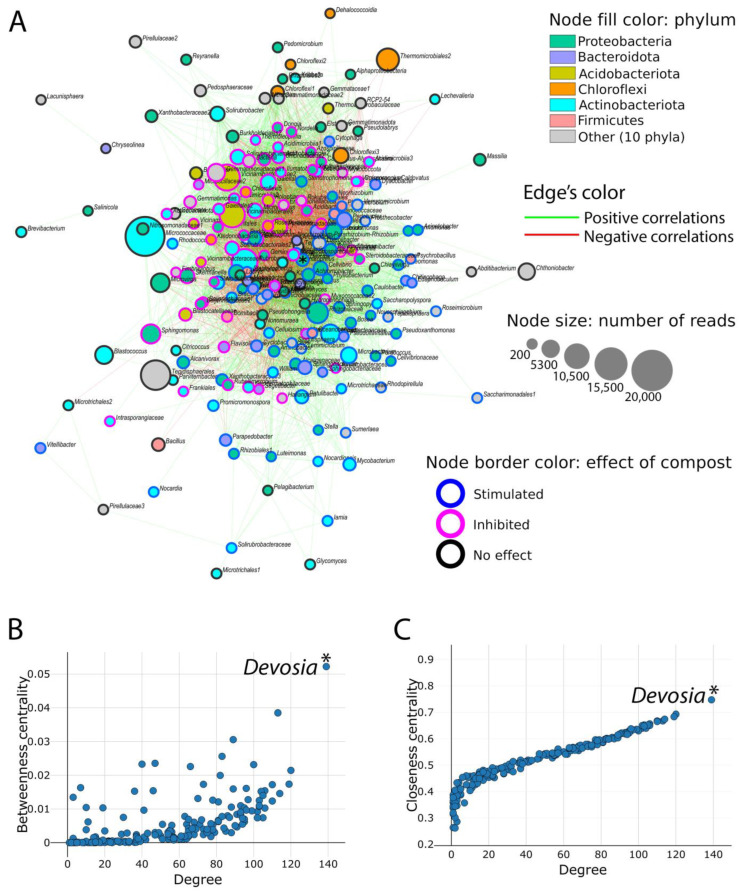
Co-occurrence network. (**A**) Network showing the significant correlations between genera based on their occurrence pattern. Only genera with a relative abundance >0.05% of total reads and occurring in at least 12 samples were included in this analysis. Compost used samples were removed from this analysis to avoid the strong habitat-preference effect, which could hide potential interactions. The node labeled with an asterisk corresponds to the genus *Devosia*, which was identified as the main network hub according to degree, betweenness centrality (**B**) and closeness centrality (**C**).

**Figure 5:**
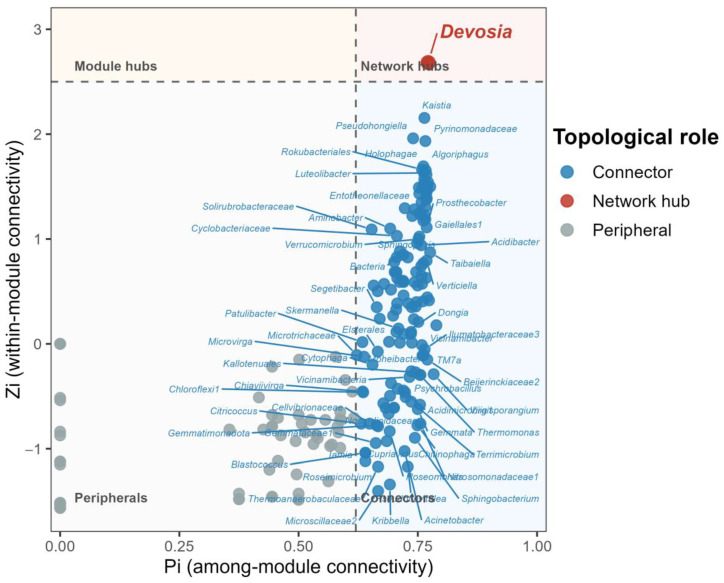
Zi/Pi plot of the co-occurrence network. The complete list of taxa with their attributes is available in the Supplementary file “Zi-Pi_results.xlsx”.

**Figure 6:**
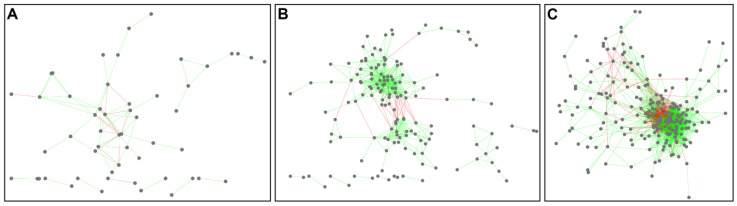
Co-occurrence networks separately generated for each treatment. (**A**) Unamended control; (**B**) MSW-compost tilled; (**C**) MSW-compost mulched. Nodes represent genus-level taxa; edges represent either positive (green lines) or negative (red lines) correlations. Networks features are reported in Table 1.

Co-occurrence analysis produced a network composed of 206 nodes and 4842 edges, with an average of 47.92 neighbors per node (Figure 4A). The genus *Devosia* was the hub taxon in this bacterial network (Figure 4A, asterisk), based on superior betweenness centrality and closeness centrality values (Figure 4B,C). Zi/Pi plots confirmed *Devosia* as the only network hub (Figure 5). The co-occurrence networks separately calculated for each treatment (compost tilled, compost mulch, and unamended control) showed a clear increase of network complexity from unamended samples (control) to MSW-compost amended samples; markedly, the mulched samples showed a much more connected network (Figure 6), with 4.3 times more total connections and 2.6 times more mean connections per node compared to tilled network, and a notable superior network heterogeneity and centralization (Table 1).

### 3.6. Taxa Differential Abundance Analysis

**Figure 7:**
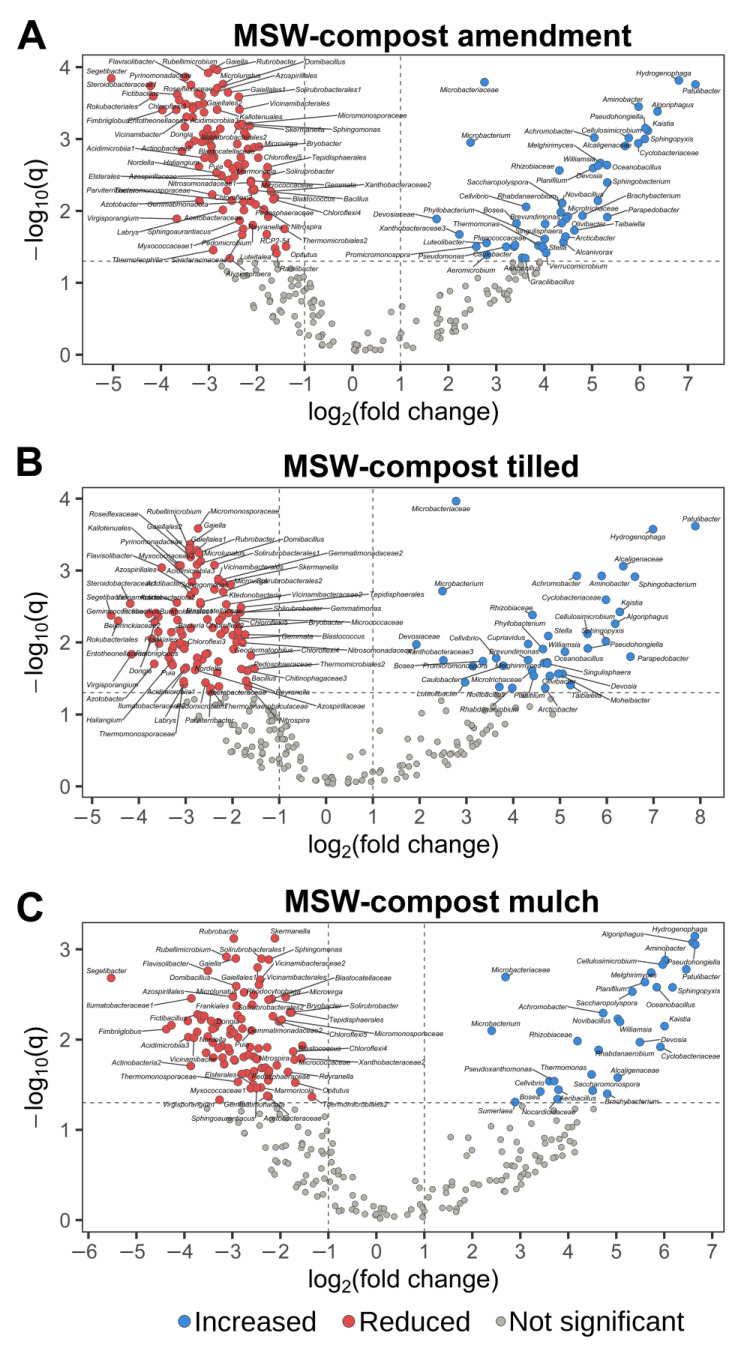
ALDEX3 plot Volcano plot, showing the response of bacterial taxa (genus-level) to MSW-compost addition. (**A**) Compost vs. No compost samples comparison; here, Compost-mulch and Compost tilled samples were merged and data from all sampling times were averaged. (**B**) Compost tilled vs. No compost samples comparison. (**C**) Compost mulch vs. No compost samples comparison.

To assess the effect of compost on the relative abundance of genus-level taxa, the samples amended with MSW-compost were merged and compared to the unamended samples, merging all sampling times (Table 2; Figure 7; Supplementary Figure S6). Additionally, Compost mulch vs. No compost and Compost tilled vs. No compost were also compared, separately (Table 2; Figure 7; Supplementary Figure S6). ALDEX3 indicated a notably higher number of significantly reduced genus-level taxa (increased/reduced ratio always <0.5, with Compost mulch giving the lower ratio; Table 2). STAMP showed a similar number of increased and reduced genus-level taxa, with ratios of 1.17, ~1 and 0.84 for Compost-amended samples (both tilled and mulch merged), Compost tilled alone, and Compost mulch alone, respectively (Table 2). In total, the number of significantly different features found was similar in the two methods, STAMP and ALDEx3 (Table 2). A further LEfSe analysis on compost-amended vs. unamended samples confirmed the results, showing a distribution more in line with the STAMP results (108 increased vs. 107 reduced taxa; Supplementary Figure S7).

Among the increased genus-level taxa, well-documented plant growth-promoters and soil health-enhancers occurred: members of the Rhizobiaceae family and related genera (*Allorhizobium-Neorhizobium-Pararhizobium-Rhizobium*, *Neorhizobium*, *Phyllobacterium*, *Aminobacter*, and *Bosea*), which suggest an enhancement of biological nitrogen fixation and root-associated mutualistic interactions. *Devosia*, in particular, the hub of the microbial network (Figure 4 and Figure 5), was under the detection limit in both the compost used and the initial soil; however, it was stimulated in the soil amended with compost. Taxa with biocontrol potential (*Pseudomonas*, *Stenotrophomonas*, *Chitinophaga*, and *Sphingobacterium*) were also increased, indicating potential improvement in the suppressiveness of soil against phytopathogens. Several taxa with recognized roles in decomposition of complex polysaccharides and mineralization of carbon and nitrogen were also increased, including *Chitinophaga*, *Cellvibrio*, *Flavobacterium*, *Dyadobacter*, *Cytophaga*, and *Edaphobaculum*. Thermotolerant Actinobacteriota, known for their lignocellulolytic and hydrolytic activities, such as *Saccharimonospora*, *Saccharopolyspora*, and *Promicromonospora*, were enriched, consistently with the thermophilic origin of the MSW-compost, as well as members of the phyla Planctomycetota (*Rhodopirellula*, *Singulisphaera*, *Tepidisphaera*, *Planctomycetales*) and Verrucomicrobiota (*Verrucomicrobium*, *Verrucomicrobiaceae*, *Prosthecobacter*), both associated with soil structural health and complex polysaccharides turnover. In contrast, the reduced taxa were mostly represented by oligotrophic and acidophilic lineages characteristic of nutrient-poor soils, including representatives of Acidobacteriota (*Vicinamibacter*, *Vicinamibacteraceae*, *Vicinamibacterales*, *Blastocatellaceae*, *Pyrinomonadaceae*, and *Holophagae*) and slow-growing actinobacterial groups (*Gaiella*, *Gaiellales*, *Thermoleophilia*, *Rubrobacter*, *Solirubrobacterales*, *Frankiales*, and *Ktedonobacteria*). Several members of bacterial-predators Myxococcota (*Myxococcaceae*, *Haliangium*) were also reduced in abundance.

Interestingly, no significantly different genus-level taxa were found between the two methods, tilled vs. mulch, of compost application; also, separating sampling times, no significantly different genus-level taxa were found.

Data were central log ratio (CLR)-transformed, and the comparison was performed with Welch’s test. Genera showing both fold change ≥2 and Benjamini-Hochberg-corrected *p* ≤ 0.05 were considered as significant. The complete list of genus-level taxa is provided in Supplementary Material (Files “ALDEx3.genera.compost_amendment.xlsx”, “ALDEx3.genera.compost_tilled.xlsx” and “ALDEx3.genera.compost_mulch.xlsx”).

### 3.7. Functional Prediction (Picrust2)

Picrust2 predicted 8734 KEGG-Orthologs and 552 functional pathways. A PCA plot based on the distances between these predicted functional pathways (Supplementary Figure S8) showed a relative positioning of the samples very similar to that obtained with genus-level taxa (Figure 3B).

To assess the effect of compost amendment on the predicted KEGG-Orthologs and functional pathways, low-abundant features (representing less than 0.001% and 0.005% of the total Picrust2-inferred number of genes, respectively) were deleted; thus, 5140 KEGG-Orthologs and 434 functional pathways remained.

**Figure 8:**
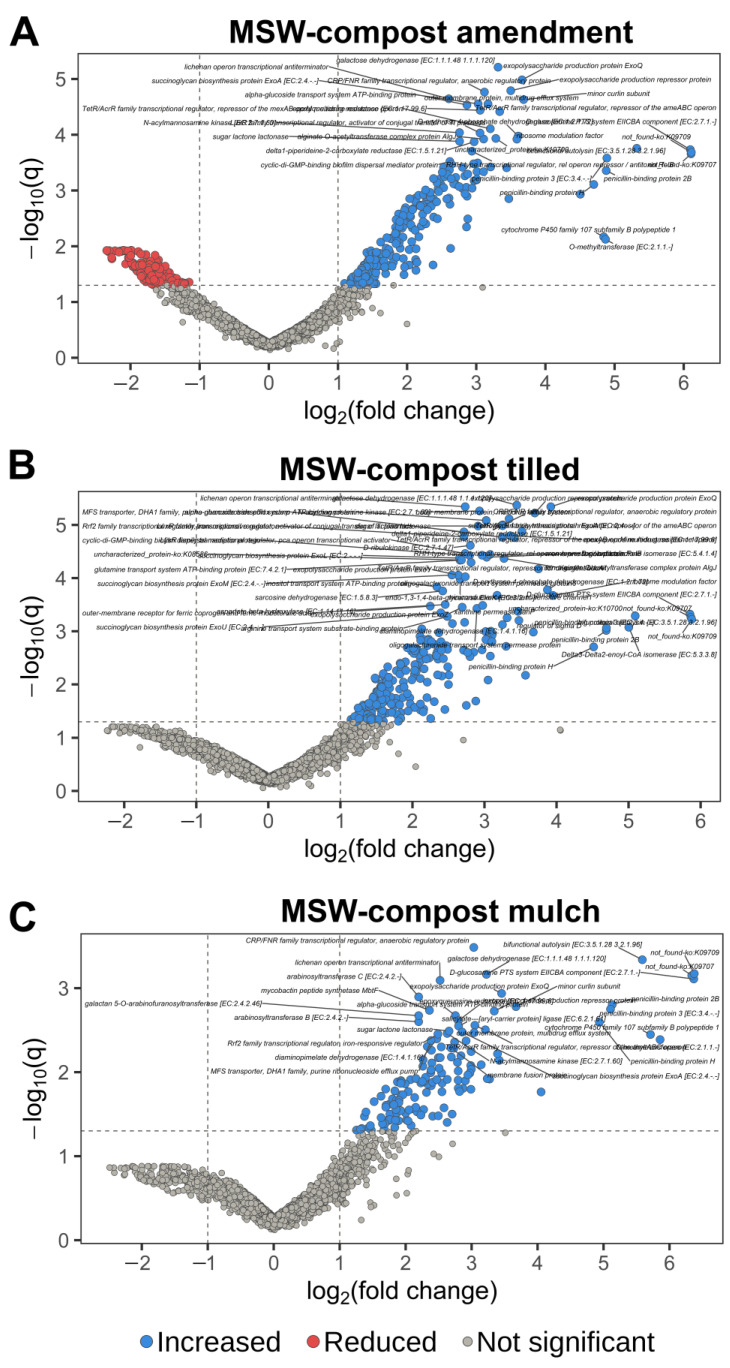
ALDEx3 Volcano plots showing the response of Picrust2’s predicted KEGG-Orthologs to MSW-compost addition. (**A**) Compost vs. No compost comparison; here, Compost-mulch and Compost tilled samples were merged and data from all sampling times were averaged. (**B**) Compost tilled vs. No compost samples comparison. (**C**) Compost mulch vs. No compost samples comparison. Data were central log ratio (CLR)-transformed, and the comparison was performed with Welch’s test. Features having minimum fold change ≥ 2 and Benjamini-Hochberg-corrected *p* ≤ 0.05 were considered as significantly affected. The complete list of KEGG-Orthologs is provided in Supplementary Material (Files “ALDEx3.Picrust2_KO.compost_amendment”, “ALDEx3.Picrust2_KO.compost_tilled” and “ALDEx3.Picrust2_KO.compost_mulch”).

**Figure 9:**
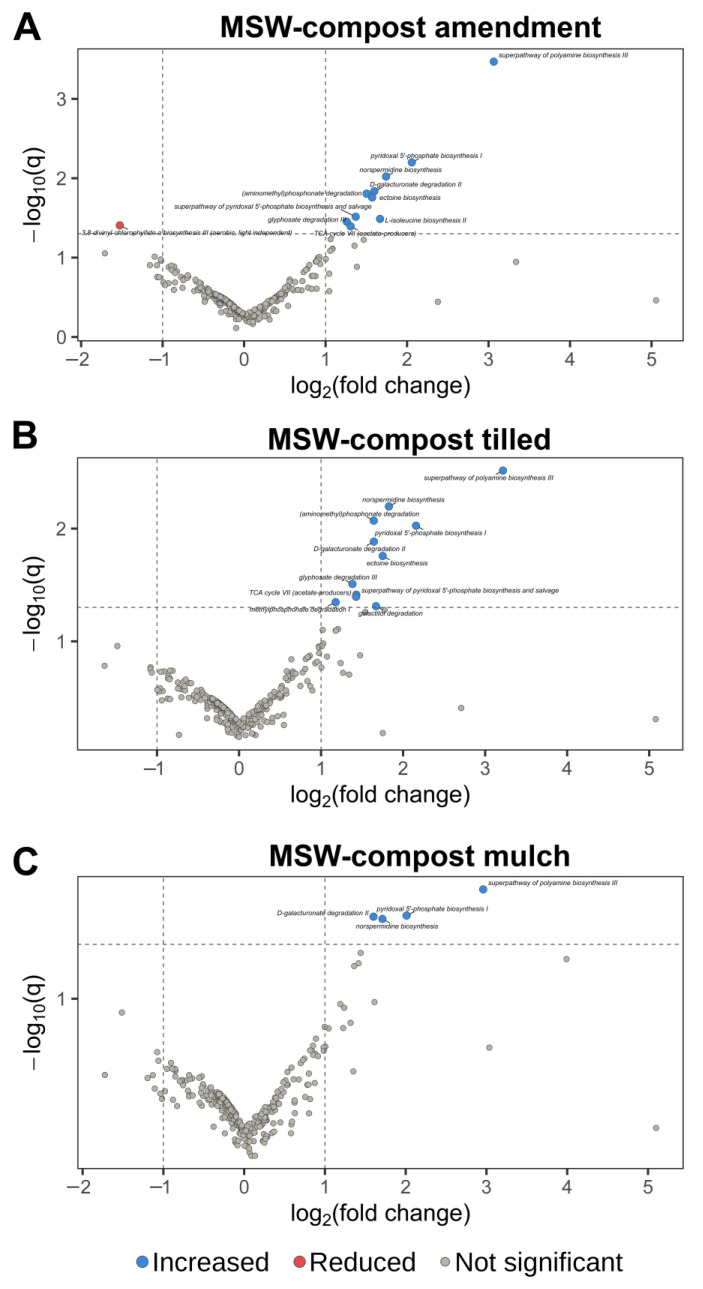
ALDEx3 Volcano plots showing the response of Picrust2’s predicted functional pathways to MSW-compost addition. (**A**) Compost vs. No compost comparison; here, Compost-mulch and Compost tilled samples were merged and data from all sampling times were averaged. (**B**) Compost tilled vs. No compost samples comparison. (**C**) Compost mulch vs. No compost samples comparison. Data were central log ratio (CLR)-transformed, and the comparison was performed with Welch’s test. Features having fold change ≥2 and Benjamini-Hochberg-corrected *p* ≤ 0.05 were considered as significantly affected. The complete list of pathways is provided in Supplementary Material (Files “ALDEx3.Picrust2_pathways.compost_amendment.xlsx”, “ALDEx3.Picrust2_pathways.compost_tilled.xlsx” and “ALDEx3.Picrust2_pathways.compost_mulch.xlsx”).

A notably higher number of these were increased by compost addition rather than reduced, particularly if considering Compost tilled and Compost mulch separately (Table 3 and Table 4; Figure 8 and Figure 9; Supplementary Figures S9 and S10). In contrast to ALDEx3, STAMP detected a notably higher number of affected features (Table 3 and Table 4) while, in the case of the taxa comparison, the number of significantly different features found was similar in the two methods (Table 2). It is worth noting the evident difference between the genus-level taxa comparison, where more reduced than increased features were assessed (Table 2) and the predicted functions comparison, where a much higher number of increased features were found (Table 3 and Table 4).

Among the increased functional pathways predicted, both biosynthesis and degradation pathways occurred (Figure 9; Supplementary Figure S10), including toluene degradation IV (aerobic, via catechol), octane oxidation, and three distinct phosphonate degradation pathways [(aminomethyl)phosphonate degradation, methylphosphonate degradation I, and glyphosate degradation III]. Additionally, pathways related to complex lipid catabolism (including plasmalogen degradation and ceramide degradation by α-oxidation), as well as ectoine biosynthesis and glycine betaine degradation I, were significantly enriched in the compost-amended soil.

Similarly to what we found for genus-level taxa, the comparison between Compost tilled and Compost mulch showed neither significantly different KEGG-Orthologs nor significantly different pathways between the two methods of compost application.

### 3.8. Tea Bag Index (TBI)

**Figure 10:**
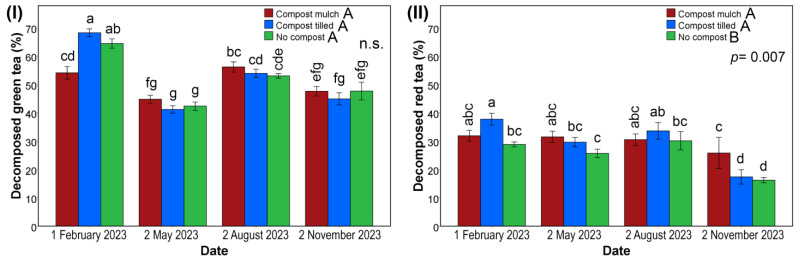
Tea Bag Index, measured as percentage of (**I**) decomposed green tea and (**II**) decomposed red tea. Different low-case letters above the bars indicate significantly different means according to one-way ANOVA followed by Duncan’s post-hoc test (*p* ≤ 0.05). Different capital letters next to the treatment names indicate significantly different means according to repeated-measures ANOVA followed by Duncan’s post-hoc test (*p*-values; n.s. = not significant). Repeated-measures ANOVA was performed on treated samples and no treated controls (sampling times T1 to T4, dates indicated), excluding the initial state (T0, both initial soil and compost used). Each bar shows the mean ± standard error of three replicates (n = 3).

**Figure 11:**
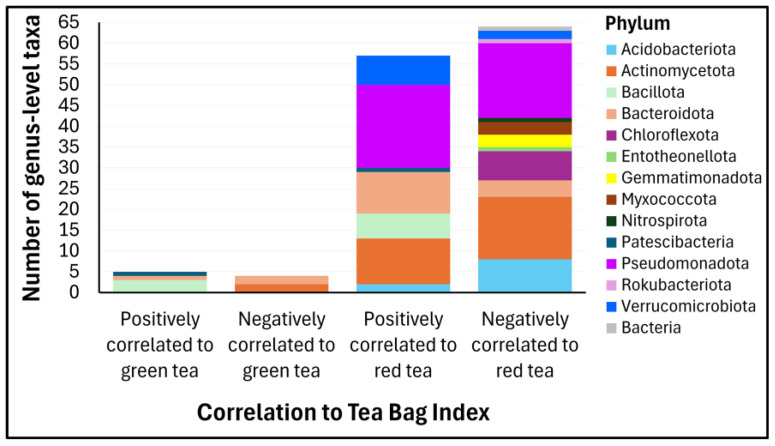
Number of significant correlations between taxa at the genus level and percentage of decomposition of green and red tea, grouped by phylum. The complete list of correlations is shown in Supplementary file “Correlations metabarcoding-TBI.xlsx”.

A significant effect of the compost amendment was found for red tea decomposition but not for green tea, which indicates a higher decomposition activity of the recalcitrant organic compounds [Figure 10I,II]. This effect was consistent for the whole period of the field experiment [Figure 10II; repeated-measures ANOVA *p* = 0.007; 1 − β = 0.87] and showed a large effect size (*η*^2^*_p_* = 0.56). Coherently, a notably higher number of genus-level taxa was found correlated with red tea compared to green tea (121 and 9, respectively; Figure 11). *Devosia*, the hub taxon of the co-occurrence network, (Figure 4 and Figure 5) was among the correlated genus-level taxa.

## 4. Discussion

In this study, we investigated, through a taxonomical approach coupled with a functional assay, the effects of the compost obtained from the organic fraction of the municipal solid waste (MSW-compost) on the soil bacterial microbiota of a productive vineyard in Southern Italy. Compost is widely used in agriculture for its fertilizing effect [55], anti-germinative properties [15,16], and soil temperature modulation when applied as mulch [11], making critical to assess how its application affects the soil microbiome, a crucial driver of soil fertility and preservation [56].

Since the appearance of MSW-compost, concerns arose about possible contaminations by human pathogens [57,58]. However, especially in recent times, increasingly stringent quality control standards have reinforced the safety of MSW-compost by ensuring that the high-temperature phase effectively kills all potentially dangerous microbes [2]. In our study, accordingly, no relevant human pathogen increase was found in the microbiota of MSW-compost-treated soil samples.

The application of MSW-compost in our trial increased the alpha-diversity indices, and this increase was maintained for the whole period of observation. This is a beneficial effect for the vineyard soil, as microbial diversity has been recognized as a key indicator of soil fertility due to the multiple ecosystem services it provides and its support to both resilience and stress mitigation [59,60]. Previous results generally showed no statistically significant effects of MSW-compost, especially for field works [61,62]. However, most of the scientific literature typically focuses on soil microbial biomass [63,64], neglecting the ecological aspects related to community changes; therefore, our study is one of the few studies that clearly indicate an increase in microbiota diversity as a consequence of MSW-compost amendment under productive field conditions.

The structure of the microbiota was affected by the addition of MSW-compost, without a difference between the two application modes, tilled or mulch. This suggests that the effects mainly depended on the chemical composition of compost more than on the physical modifications that might derive from MSW-compost incorporation into the soil. Interestingly, one year after MSW-compost amendment, the structure of the microbiota still differed significantly from that of unamended soil, although the effect size decreased over time. The temporal dynamics observed in our study are consistent with the response known to induce a strong initial shift in soil microbial communities, followed by a milder and more sustained response over time [65]. This decline in treatment effects over time has been linked to the breakdown of more labile compost fractions and the gradual re-establishment of the soil’s native microbial networks [65]; however, comparing these results with our observation, it can be argued that the stimulated microbiota, one year after treatment, was the one specialized in recalcitrant compounds metabolism. The effects of compost on the soil microbiome depend on several factors (soil management history, baseline microbial diversity, organic matter content, etc.); a single addition of compost was shown to affect microbiota structure and persisted until the 227th day after application [66]. Although data on MSW-compost are quite scarce, significant effects of repeated MSW-compost addition on microbial biomass for up to nine years were found [67], whereas in another study, 44% of the soil bacterial community was affected by compost addition [68]. Sadet-Bourgeteau et al. [69] showed a dose-dependent effect of organic waste products on soil microbiota. A recent field study [70] confirmed that MSW-compost promoted plant-beneficial microbial taxa in the wheat rhizosphere in a single growing season; however, longitudinal studies tracking dynamics of soil microbial responses to MSW-compost over multiple timepoints remain underrepresented in the literature, highlighting the relevance of this study. As a future perspective, it would be useful to determine whether this difference persists across subsequent growing seasons or gradually diminishes and disappears, as this trend could help inform farmers on the optimal frequency of compost application.

The hub taxon (as resulting from the connectivity within the co-occurrence network) appeared to be the genus *Devosia*, which was also increased by MSW-compost addition, regardless of the application mode; so far, this genus has been rarely retrieved in studies dealing with soil- and crop-associated microbiota, with a few exceptions. For example, Han et al. [71] also identified *Devosia* as the key species in the microbial network. Wei et al. [72] found *Devosia* among the taxa enriched after compost application in alfalfa rhizosphere, while Chang et al. [73] reported an increase of *Devosia* after biogas residue composting and showed that it was positively correlated with humic acid produced. In a recent study, it was shown that, after 60 days of composting winery residues, *Devosia* (initially not detected) represented the fifth more frequent taxon [74]. All this evidence, in light of our observations, could indicate that there is a link between *Devosia*, the composting process, and the grapevine environment. *Devosia*, as further demonstrated by a few studies, can harbor plant growth promoting properties like auxin and siderophore production [75]. Soil inoculation of a cell-free supernatant obtained from a *Devosia* sp. isolate from root nodules increased canola and soybean growth up to +200% under saline conditions [76]. The stimulation of *Devosia* in the MSW-compost amended vineyard soil suggests a latent potential for growth promotion and tolerance to abiotic stresses that deserves further in planta investigation.

Interestingly, the only difference detected in our study between the two modes of MSW-compost application (tilled or mulched) concerned the connectivity level of their co-occurrence networks: the application as mulch led to a much more complex and denser microbial network, with all nodes connected within a unique module and a much higher average degree compared to both unamended control and tilled application. These interesting results highlight how the mulched application of MSW-compost can exert more benefits than its incorporation in soil by tillage: in fact, a higher connectivity of the microbial network has been linked to healthier soils and enhanced microbiome stability [77]. The reasons for this difference might be related to the protective effects exerted by mulching (such as protection from solar radiation, retention of soil humidity, preservation of soil temperature homeostasis, etc.) [11,12].

Our results showed that MSW-compost amendment exerted a strong selective pressure on the soil bacterial community, broadly favoring copiotroph-associated and functionally active taxa at the expense of oligotroph taxa: this pattern aligns with the established ecological theory of *r*/K-strategy trade-offs in soil microbiology, whereby inputs of labile and complex organic carbon stimulate fast-growing, metabolically versatile organisms while reducing slow-growing, oligotrophic specialists adapted to nutrient-limited conditions [78]. Notably, the enrichment of multiple genera of the Rhizobiales order suggests that compost application may enhance the capacity of the microbiota to both support biological nitrogen fixation and reinforce mutualistic interactions with plant roots. Similarly, the stimulation of chitinolytic and cellulolytic taxa, many of which also produce secondary metabolites with antifungal activity, points to a dual improvement in nutrient availability and disease suppression potential, two key components of soil ecosystem services in agroecosystems. The consistent inhibition of Acidobacteriota subgroups, especially the Vicinamibacterales order, can be explained by their tendency to decline when the availability of organic substrates in soil increases, as typically observed following organic amendment [79]. From a methodological point of view, the results delivered from ALDEx3 and STAMP showed a difference, as STAMP indicated a higher number of increased taxa (82 vs. 70), while ALDEx3 indicated a prevalent reduction (49 vs. 102). Moreover, we noticed that STAMP did not consider 22 taxa with zero frequency in the control group (unamended soil), ten of which were detected as significantly increased by ALDEx3. Therefore, it appears that, due to the CLR transformation, ALDEx3 is able to deliver more reliable results. Indeed, LEfSe analysis was more in line with the results of STAMP (both methods do not use CLR transformation to account for data compositionality). This highlights the importance of not relying on one single tool, but substantiating the results with multiple approaches instead.

In line with the shift in taxa composition, functional prediction showed a significant response of the soil microbiota to MSW-compost addition in terms of functional shift; however, while a similar number of taxa were both increased and decreased, the functions were largely increased, suggesting that MSW-compost application specifically increases the metabolically active members of the soil microbiota. Both degradative and synthetic pathways were enriched and, together with the Tea Bag Index (TBI) results (significantly increased capacity to degrade red tea), these data provide substantial evidence toward the increased capacity of the soil microbiota in degrading recalcitrant organic compounds. The enrichment of multiple catabolic pathways in the MSW-compost amended soil is sustained by both the chemical composition of MSW-compost and its associated microbes. The simultaneous enrichment of toluene degradation via catechol and octane oxidation pathways indicates an enhanced potential of aromatic and aliphatic hydrocarbon catabolism in the amended soil. MSW-compost is a source of organic compounds, including both polycyclic aromatic and aliphatic hydrocarbons, derived from household waste inputs like plastic-based packaging residues, lubricants, and personal care products [80]. These compounds likely exerted a selective pressure on the microbiota, favoring the establishment of hydrocarbon-degrading taxa [81]. Under our experimental conditions, the results of the TBI showed a significantly higher red tea (rich in polyphenols, flavonoids, and other aromatic structures) mass loss in the amended soil, indicating that the predicted enrichment of recalcitrant compound-degraders translated into actual decomposition activity: in fact, the catechol-mediated aromatic ring cleavage pathway is mechanistically relevant not only to hydrocarbon degradation but also to the breakdown of both polyphenolic and lignin-derived compounds [82].

The enrichment of three phosphonate degradation pathways (aminomethylphosphonate, methylphosphonate, and glyphosate) in MSW-compost amended soil samples represents an interesting diagnostic signal: phosphonates, in fact, are organophosphorus compounds characterized by very stable C–P bonds, and their mineralization is metabolically costly [83]. Their presence in MSW-compost could be attributed either to glyphosate residues in plant-derived waste or to phosphonate-based industrial chelating agents commonly found in household cleaning products, which resulted in an enrichment of bacteria harboring C–P lyases. The enrichment of these pathways indicates the intrinsic resilience and bioremediation capacity of the microbiome stimulated by the MSW-compost, acting as an ecological filter that prevents the accumulation of these compounds in the vineyard ecosystem and their translocation to the vine plants. The enrichment of both plasmalogen and ceramide degradation pathways indicates an enhanced capacity to mineralize refractory lipid structures (among the more recalcitrant components of soil organic matter), consistent with the input of animal-derived residues (meat, fish, dairy products, etc.) typically occurring in the organic fraction of municipal solid waste [84]. The presence of both ectoine biosynthesis and glycine betaine degradation pathways in the MSW-compost amended soil is consistent with a legacy effect of compost-inhabiting microbiota: both compounds, in fact, play a role as osmoprotectants under both osmotic and drought stress, conditions that are commonly occurring during the composting process [85]. Bacteria pre-adapted to these stresses during composting, once introduced into the soil with the amendment, appear to maintain their functional signature over time. Both ectoine and glycine betaine play roles in cellular protection and response to osmotic stresses in both microbes and plants; therefore, their enrichment can positively foster vineyard soil resilience and support grapevine resistance to abiotic stresses [86]. Notably, stress-tolerant bacterial taxa have been repeatedly associated with enhanced degradation of recalcitrant organic substrates in soil [87].

The complex chemical mixture of MSW-compost provides both readily available carbon (stimulating copiotrophic taxa) and recalcitrant substrates selecting for specialized degraders, some of which derive from the compost itself while others are specifically stimulated from the original soil microbiota reservoir. A dedicated study might elucidate these dynamics in relation to soil biodiversity and development, also taking into account relevant enzymatic activities (e.g., dehydrogenase, urease, phosphatase, β-glucosidase).

Interestingly, the comparison between the mode of MSW-compost application (tilled vs. mulch) showed significant differences only in terms of network connectivity, whereas neither taxonomical composition nor functionality were affected. This is a notable result, since, to the best of our knowledge, no previous study has directly compared the effect of compost application mode on soil microbiota. The most similar work [88] compared, through Denaturing Gradient Gel Electrophoresis and Community-Level Catabolic Profile, tillage vs. surface mulching of different organic amendments (including composted cotton gin trash) and found no statistically significant effect of application method on bacterial community diversity, with differences driven primarily by amendment type rather than mode of application.

In our study, we did not measure the physicochemical parameters of the MSW-compost amended soils, as the main focus was on microbial diversity and functionality. Although this appears to be an evident limitation, it should be noted that there is a large body of literature dealing with this topic, which has consistently shown that compost addition to soil increases the content of both macro- and micro-nutrients [89,90], which is also logical considering the chemical composition of composts. These expected effects of compost on soil chemistry are in agreement with our observations on microbial composition, activity, and network.

## 5. Conclusions and Outlook

Our study demonstrated that amendment with MSW-compost had a positive effect on vineyard soil, as it led to increased microbial diversity, functionality, and network connectivity, especially when applied as mulching. Metabolically active microbial taxa were particularly enriched, which suggests that MSW-compost acts not only as a potential bioinoculant, but also as a driver of passive biostimulation: substrate-driven community selection and direct microbial inoculation jointly enhance the decomposition activity of recalcitrant organic matter in soil. While this may be interpreted positively in terms of increased microbial diversity and organic matter turnover, it also raises questions about the long-term accumulation of xenobiotics and trace organic contaminants in amended soils, warranting further investigation through physicochemical characterization of MSW-compost amended agricultural soils. Future investigations and follow-up studies should combine bacterial and fungal analysis to obtain a holistic view of microbiome responses to MSW-compost amendment in the longer term, also considering the interactions with the agronomic practices.

## Figures and Tables

**Table 1:** Comparison of network features based on the co-occurrence networks generated for each treatment separately (see Figure 6).

Network Feature	Unamended Control	MSW-Compost Tilled	MSW-Compost Mulch
Number of nodes	53	149	213
Number of edges	68	528	2306
Avg. number of neighbors	3.533	8.311	21.653
Clustering coefficient	0.285	0.430	0.491
Network density	0.122	0.069	0.102
Network heterogeneity	0.704	0.889	1.037
Network centralization	0.276	0.258	0.335
Connected components	10	10	1

**Table 2:** Number of bacterial genus-level taxa significantly affected by compost addition, according to Welch’s test with Benjamini-Hochberg corrected *p* ≤ 0.05 and fold change ≥ 2 (performed in the software STAMP). In ALDEx3, the same statistical test and the same thresholds for significance were used, but the data were initially central log ratio (CLR)-transformed. For this analysis, 276 genus-level taxa with relative abundance > 0.05% of total reads were used, and all sampling times were merged.

Comparison	Genus-Level Taxa Increased by MSW-Compost		Genus-Level Taxa Reduced by MSW-Compost		Increased/Reduced Ratio	
	STAMP	ALDEx3	STAMP	ALDEx3	STAMP	ALDEx3
All compost-treated samples vs. No compost	82	49	70	102	1.17	0.48
Compost tilled vs. No compost	75	40	74	96	1.01	0.42
Compost mulch vs. No compost	58	31	69	93	0.84	0.33

**Table 3:** Number of KEGG-Orthologs and functional pathways, as predicted by Picrust2, significantly affected by compost addition, according to Welch’s test with Benjamini-Hochberg corrected *p* ≤ 0.05 and fold change ≥ 2 (performed in the software STAMP). In ALDEx3, the same statistical test and the same thresholds for significance were used, but the data were initially central log ratio (CLR)-transformed. For this analysis, 5140 KEGG-Orthologs with a relative abundance > 0.001% of total predicted number of gene copies were used, and all sampling times were merged.

Comparison	KEGG-Orthologs Increased by MSW-Compost		KEGG-Orthologs Reduced by MSW-Compost		Increased/Reduced Ratio	
	STAMP	ALDEx3	STAMP	ALDEx3	STAMP	ALDEx3
All compost-treated samples vs. No compost	580	253	108	113	5.37	2.24
Compost tilled vs. No compost	589	255	124	0	4.75	∞
Compost mulch vs. No compost	545	141	103	0	5.29	∞

**Table 4:** Number of functional pathways, as predicted by Picrust2, significantly affected by compost addition, according to Welch’s test, with Benjamini-Hochberg corrected *p* ≤ 0.05 and fold change ≥ 2 (performed in the software STAMP). In ALDEx3, the same statistical test and the same thresholds for significance were used, but the data were initially central log ratio (CLR)-transformed. For this analysis, 434 Functional pathways with a relative abundance > 0.005% of total predicted number of gene copies were used, and all sampling times were merged.

Comparison	Functional Pathways Increased by MSW-Compost		Functional Pathways Reduced by MSW-Compost		Increased/Reduced Ratio	
	STAMP	ALDEx3	STAMP	ALDEx3	STAMP	ALDEx3
All compost-treated samples vs. No compost	37	10	2	1	18.5	10
Compost tilled vs. No compost	37	11	2	0	18.5	∞
Compost mulch vs. No compost	34	4	1	0	34	∞

## Data Availability

The data presented in this study are openly available in European Nucleotide Archive at https://www.ebi.ac.uk/ena, reference number PRJEB112833.

## Supplementary Materials

The following supporting information can be downloaded at: https://www.mdpi.com/article/10.3390/microorganisms14061372/s1, Figure S1—Pictures of the MSW-compost used and the experimental vineyard; Figure S2—Experimental design of the field trial; Table S1—Physicochemical parameters of the MSW-compost used in this work; Figure S3—Good’s coverage calculated on the ASV table at genus level; Figure S4—PCA plot showing the relative distances between all samples, including Compost used samples; Figure S5—Temporal trajectories of centroids per treatment; Table S2—Bray-Curtis dissimilarities of the compost-treated samples’ centroids to the No compost samples’ centroids; Figure S6—STAMP Extended error bar plot of genus-level taxa; Figure S7—LEfSe analysis of genus-level taxa; Figure S8—STAMP PCA plot of Picrust2’s predicted functional pathways; Figure S9—STAMP Extended error bar plots of Picrust2 KEGG-Orthologs; Figure S10—STAMP Extended error bar plots of Picrust2 Functional pathways; Excel files—“Zi-Pi_results.xlsx”, STAMP.genera.compost_amendment.xlsx”, “STAMP.genera.compost_tilled.xlsx”, “STAMP.genera.compost_mulch.xlsx”, “STAMP.Picrust2_KO.compost_amendment.xlsx”, “STAMP.Picrust2_KO.compost_tilled.xlsx”, “STAMP.Picrust2_KO.compost_mulch.xlsx”, “STAMP.Picrust2_pathways.compost_amendment.xlsx”, “STAMP.Picrust2_pathways.compost_tilled.xlsx”, “STAMP.Picrust2_pathways.compost_mulch.xlsx”, “ALDEx3.genera.compost_tilled.xlsx”, “ALDEx3.genera.compost_mulch.xlsx”, “ALDEx3.genera.compost_amendment.xlsx”, “ALDEx3.Picrust2_KO.compost_amendment.xlsx”, “ALDEx3.Picrust2_KO.compost_tilled.xlsx”, “ALDEx3.Picrust2_KO.compost_mulch.xlsx” “ALDEx3.Picrust2_pathways.compost_amendment.xlsx”, “ALDEx3.Picrust2_pathways.compost_tilled.xlsx”, “ALDEx3.Picrust2_pathways.compost_mulch.xlsx”, “LEfSE.genera.compost_amendment.xlsx”, “Correlations metabarcoding-TBI.xlsx”.

## Abbreviations

The following abbreviations are used in this manuscript:

ASV	Amplicon Sequence Variant
BC	Bray-Curtis
CLR	Central Log Ratio
FDR	False Discovery Rate
KEGG	Kyoto Encyclopedia of Genes and Genomes
MSW	Municipal Solid Waste
PCR	Polymerase Chain Reaction
PGPR	Plant Growth-Promoting Rhizobacteria
TBI	Tea Bag Index
