# Supplementary material for: Municipal Solid Waste (MSW)-Compost Amendment Increases Diversity, Functional Activities, and Network Connectivity of a Vineyard Soil Microbiota
Source: Microorganisms. 2026 Jun 21;14(6):1372. doi: 10.3390/microorganisms14061372 (PMC13305556; doi:10.3390/microorganisms14061372)
Supplement: Supplementary file 1 [file microorganisms-14-01372-s001.zip › microorganisms-4345652_Supplementary material_proofs.pdf]

Municipal solid waste (MSW)-compost amendment increases diversity, functional activities, and network connectivity of a vineyard soil microbiota.

Supplementary material

- **Figure S1** – (A) The MSW-compost used in this work, positioned next to the experimental vineyard. (B) The experimental vineyard soil after MSW-compost application (treatments are indicated with white arrows).
- **Figure S2** – Experimental design of the field trial. Treatments are indicated as labels. Grey fill indicates the unanalyzed border blocks, whereas color fill indicates the analyzed blocks (brown: Compost mulch; blue: Compost tilled; green: No compost).
- **Table S1** – Physicochemical parameters of the MSW-compost used in this work. Report of municipal solid waste compost provided by Heracle S.r.l. (Erchie, Italy). Sampling procedures = UNI 4 EN 12579:2014. Operator =Chemicalab (Modena, Italy). Date of analysis: 30 September 2022.
- **Figure S3** – A) Good's coverage calculated on the ASV table at genus level (QIIME2's taxonomical level 6). B) Magnification showing that, at the rarefaction depth (10,670 reads per sample), all samples showed a coverage >99%.
- **Figure S4** – PCA plot showing the relative distances between all samples, including Compost used samples. Samples are colored according to the treatment (base colors) and to the sampling times (color tones).
- **Figure S5** – Temporal trajectories of centroids per treatment.
- **Table S2** – Bray-Curtis dissimilarities of the compost-treated samples' centroids to the No_compost samples' centroids.
- **Figure S6** – STAMP Extended error bar plot, showing the response of bacterial taxa (genus-level) to MSW-compost addition. A) Compost *vs.* No compost samples comparison; here, Compost-mulch and Compost-tilled samples were merged and data from all sampling times were averaged. B) Compost tilled *vs.* No compost samples comparison. C) Compost mulch *vs.* No compost samples comparison. The comparison was performed with Welch's test. Genera

showing both fold change ≥2 and Benjamini-Hochberg-corrected *p*≤0.05 were considered as significant. Error bars indicate 95% confidence interval. Only significantly different taxa were plotted. The complete list of genus-level taxa is provided in Supplementary material (files "STAMP.genera.compost_amendment.xlsx", "STAMP.genera.compost_tilled.xlsx", and "STAMP.genera.compost_mulch.xlsx").

- **Figure S7** – LEfSe plot showing the response of bacterial taxa (genus-level) to MSW-compost addition (Compost *vs.* No compost samples comparison; Compost-mulch and Compost-tilled samples merged and data from all sampling times were averaged). Genera showing both Linear Discriminant Analysis Effect Size ≥2 and Benjamini-Hochberg-corrected *p*<0.05 were considered as significant. Only significantly different taxa were plotted. The list of these significant genus-level taxa is provided in Supplementary material (files "LEfSe.genera.compost_amendment.xlsx").
- **Figure S8** – STAMP PCA plot of Picrust2's predicted functional pathways, showing the relative distances between samples, colored according to treatment (base colors) and sampling time (color tones). Compost used samples were removed due to their extreme distance which would flatten all other samples.
- **Figure S9** – STAMP Extended error bar plots showing the response of Picrust2's predicted KEGG Orthologs to MSW-compost addition. A) Compost *vs.* No compost comparison; here, Compost-mulch and Compost-tilled samples were merged and data from all sampling times were averaged. B) Compost tilled *vs.* No compost samples comparison. C) Compost mulch *vs.* No compost samples comparison. The comparison was performed with Welch's test. Features having minimum fold change ≥2 and Benjamini-Hochberg-corrected *p*≤0.05 were considered as significantly affected. Error bars indicate 95% confidence interval. Only significantly different KEGG orthologs are plotted. The complete list of genus-level taxa is provided in Supplementary material (files "STAMP.Picrust2_KO.compost_amendment.xlsx", "STAMP.Picrust2_KO.compost_tilled.xlsx" and "STAMP.Picrust2_KO.compost_mulch.xlsx").
- **Figure S10** – STAMP Extended error bar plots showing the response of Picrust2's predicted Functional pathways to MSW-compost addition. A) Compost *vs.* No compost comparison; here, Compost-mulch and Compost-tilled samples were merged and data from all sampling times were averaged. B) Compost tilled *vs.* No compost samples comparison. C) Compost mulch *vs.* No compost samples comparison. The comparison was performed with Welch's test. Features having minimum fold change ≥2 and Benjamini-Hochberg-corrected *p*≤0.05

were considered as significantly affected. Error bars indicate 95% confidence interval. Only significantly different Functional pathways are plotted. The complete list of genus-level taxa is provided in Supplementary material (files "STAMP.Picrust2_pathways.compost_amendment.xlsx", "STAMP.Picrust2_pathways.compost_tilled.xlsx" and "STAMP.Picrust2_pathways.compost_mulch.xlsx").

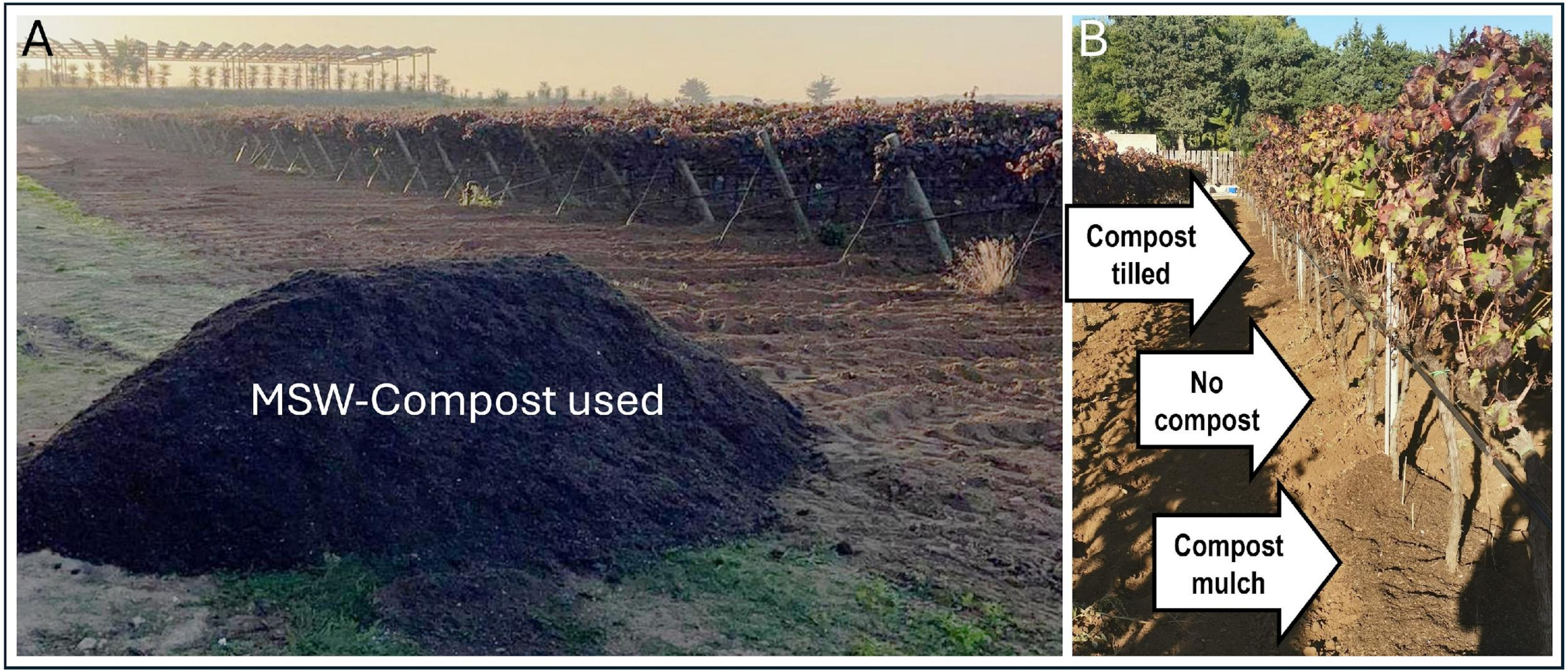

Supplementary Figure S1. (A) The MSW-compost used in this work, positioned next to the experimental vineyard. (B) The experimental vineyard soil after MSW-compost application (treatments are indicated with white arrows).

**No compost**	**Compost mulch**	**Compost tilled**	**No compost**	**No compost**	**Compost mulch**	**Compost tilled**	**Compost tilled**	**No compost**	**Compost mulch**	**No compost**
**No compost**	**Compost tilled**	**No compost**	**Compost mulch**	**Compost mulch**	**Compost tilled**	**No compost**	**No compost**	**Compost mulch**	**Compost tilled**	**No compost**
**No compost**	**No compost**	**Compost mulch**	**Compost tilled**	**Compost tilled**	**No compost**	**Compost mulch**	**Compost mulch**	**Compost tilled**	**No compost**	**No compost**

Supplementary Figure S2. Experimental design of the field trial. Treatments are indicated as labels. Grey fill indicates the unanalyzed border blocks, whereas color fill indicates the analyzed blocks (brown: Compost mulch; blue: Compost tilled; green: No compost).

Supplementary Table S1. Physicochemical parameters of the MSW-compost used in this work. Report of municipal solid waste compost provided by Heracle S.r.l. (Erchie, Italy). Sampling procedures = UNI 4 EN 12579:2014. Operator =Chemicalab (Modena, Italy). Date of analysis: 30 September 2022.

**Parameter**	**Value**	**Measure unit**
Ammoniacal nitrogen	0.309 ±0.031	% dw
Organic nitrogen	1.61 ±0.16	% dw
Organic nitrogen (% N_{Tot})	83.9 ±8.4	% N_{Tot}
Total nitrogen	1.92 ±0.34	% dw
Organic carbon	37.5 ±3.8	% dw
Fulvic and Humic Carbon	11.7 ±1.2	% dw
Plastic, glasses, metals (fraction ≥2mm)	<0.1	% dw
Inert lithoids	<0.1	% dw
Cadmium (Cd)	<0.5	mg/Kg dw
Chromium VI (Cr VI)	<0.25	mg/Kg dw
Total Chromium (Cr)	8.21	mg/Kg dw
Mercury (Hg)	<0.2	mg/Kg dw
Nichel (Ni)	8.39 ±0.84	mg/Kg dw
Lead (Pb)	13.3 ±1.3	mg/Kg dw
Copper (Cu)	65.3 ±6.5	mg/Kg dw
Thallium (Tl)	<0.5	mg/Kg dw
Zinc (Zn)	190 ±19	mg/Kg dw
pH	7.14 ±0.44	
N_{org}/N_{tot}	0.84 ±0.17	
C/N	19.5 ±2.0	
Dry matter	64.4 ±6.4	%
Salinity	61 ±12	meq/100
Electrical conductivity	4878 ±980	µS/cm
Total humidity	35.6 ±7.1	%
Escherichia coli	0	CFU/g
Salmonella spp.	Absent	In 25 g

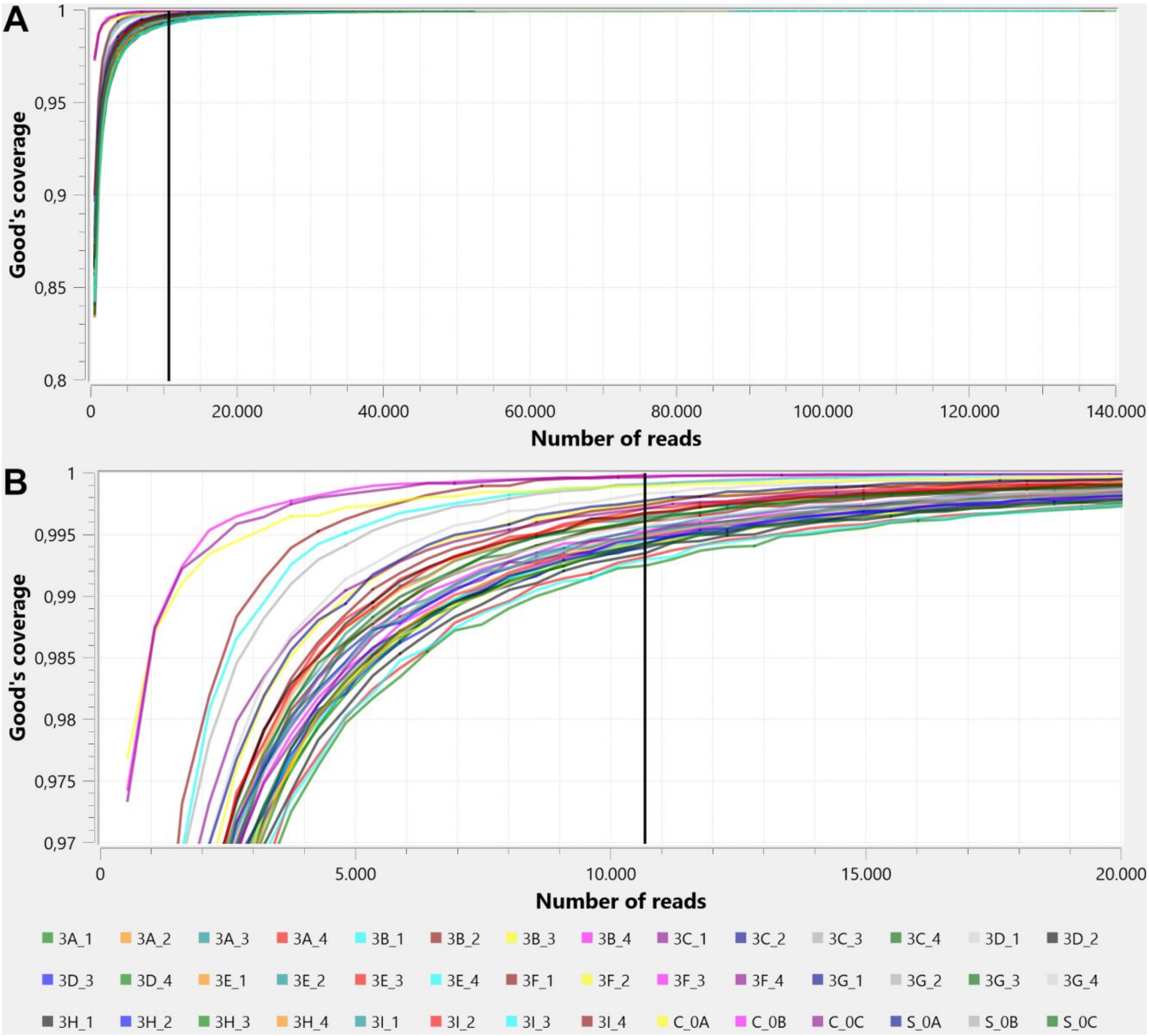

Supplementary Figure S3. A) Good's coverage calculated on the ASV table at genus level (QIIME2's taxonomical level 6). B) Magnification showing that, at the rarefaction depth (10,670 reads per sample), all samples showed a coverage >99%.

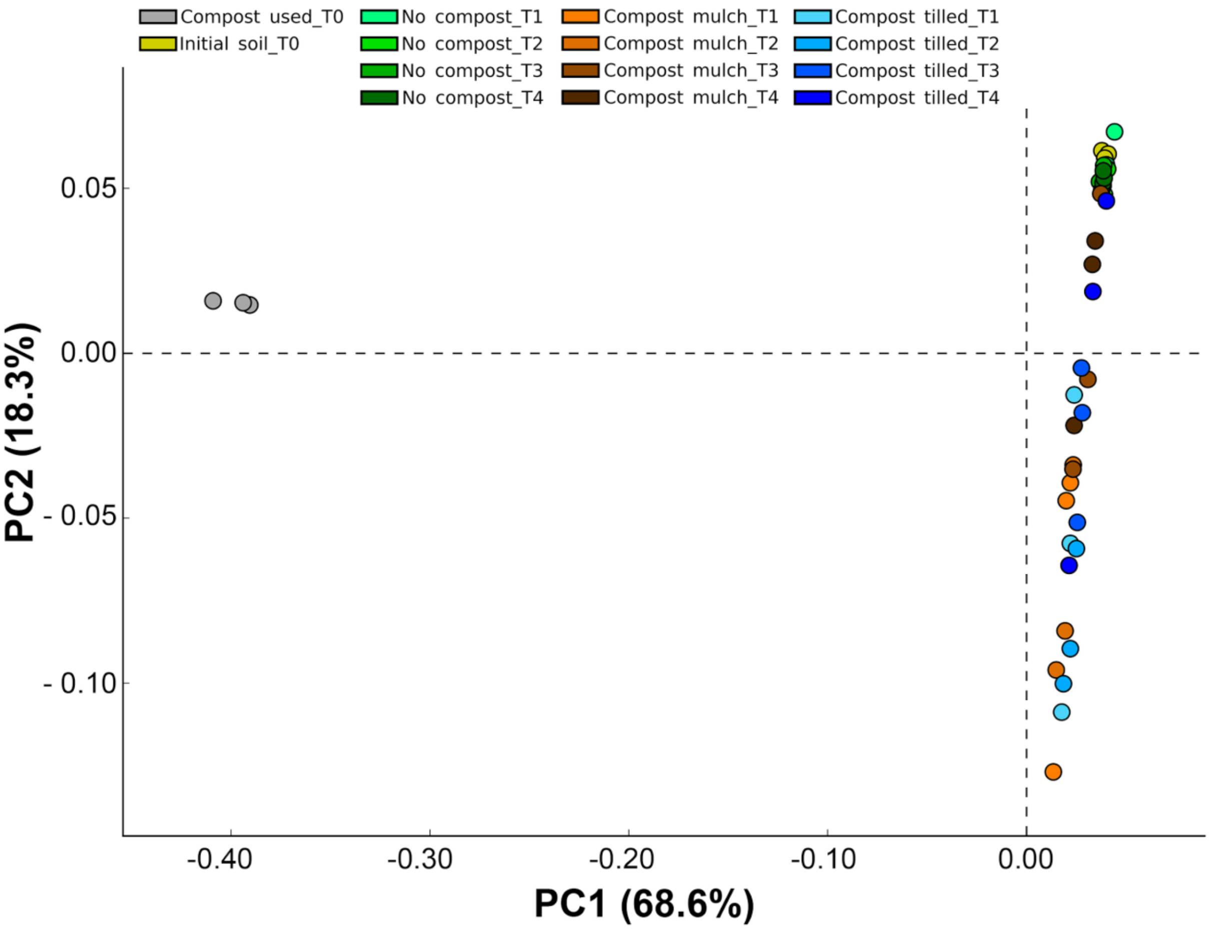

Supplementary Figure S4. PCA plot showing the relative distances between all samples, including Compost used samples. Samples are colored according to the treatment (base colors) and to the sampling times (color tones).

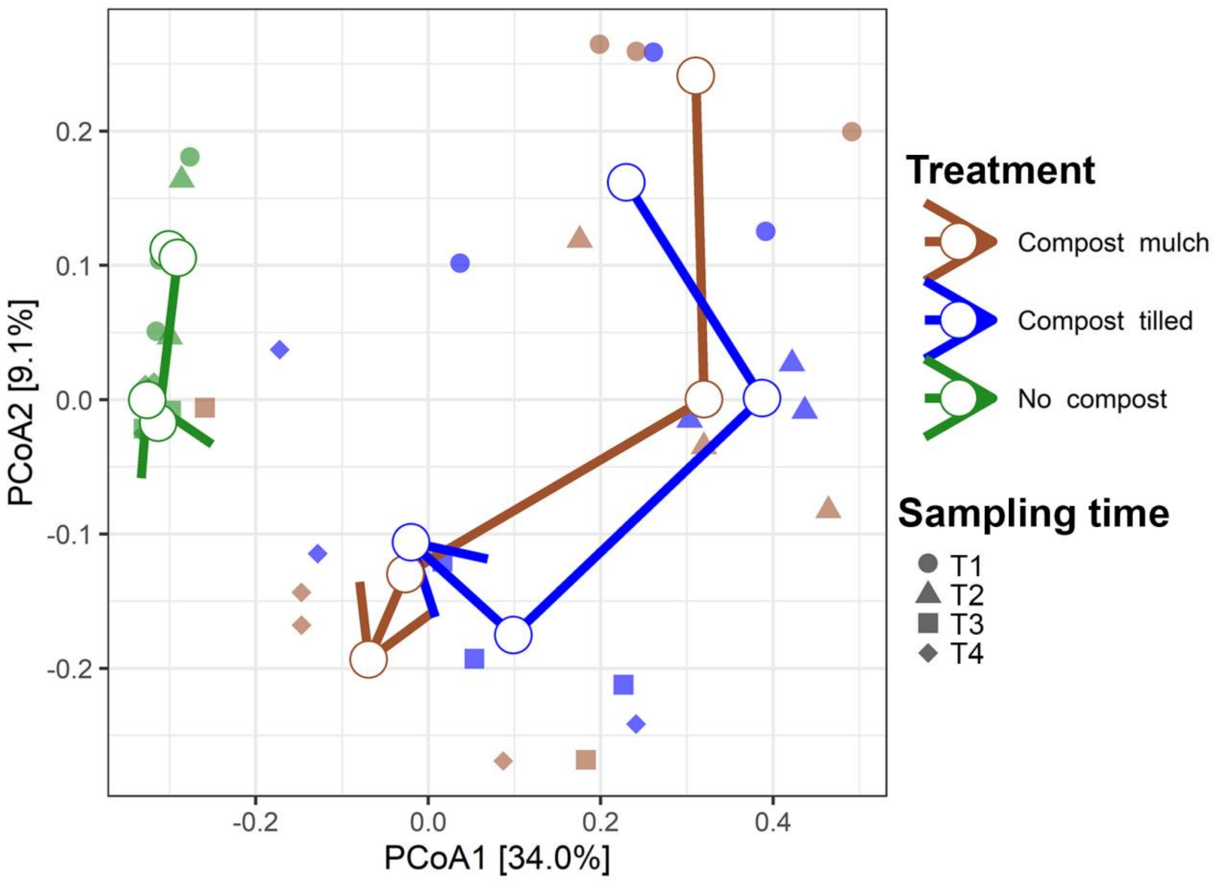

Supplementary Figure S5. Temporal trajectories of centroids per treatment.

Supplementary Table S2. Bray-Curtis (BC) dissimilarities from compost-treated samples' centroids to No compost samples' centroids.

Treatment	Sampling time	BC dissimilarities to No compost centroid
Compost_mulch	T1 (Feb. 1st, 2023)	0.625
Compost_mulch	T2 (May 2nd, 2023)	0.619
Compost_mulch	T3 (Aug. 2nd, 2023)	0.308
Compost_mulch	T4 (Nov. 2nd, 2023)	0.321
Compost_tilled	T1 (Feb. 1st, 2023)	0.533
Compost_tilled	T2 (May 2nd, 2023)	0.686
Compost_tilled	T3 (Aug. 2nd, 2023)	0.441
Compost_tilled	T4 (Nov. 2nd, 2023)	0.324

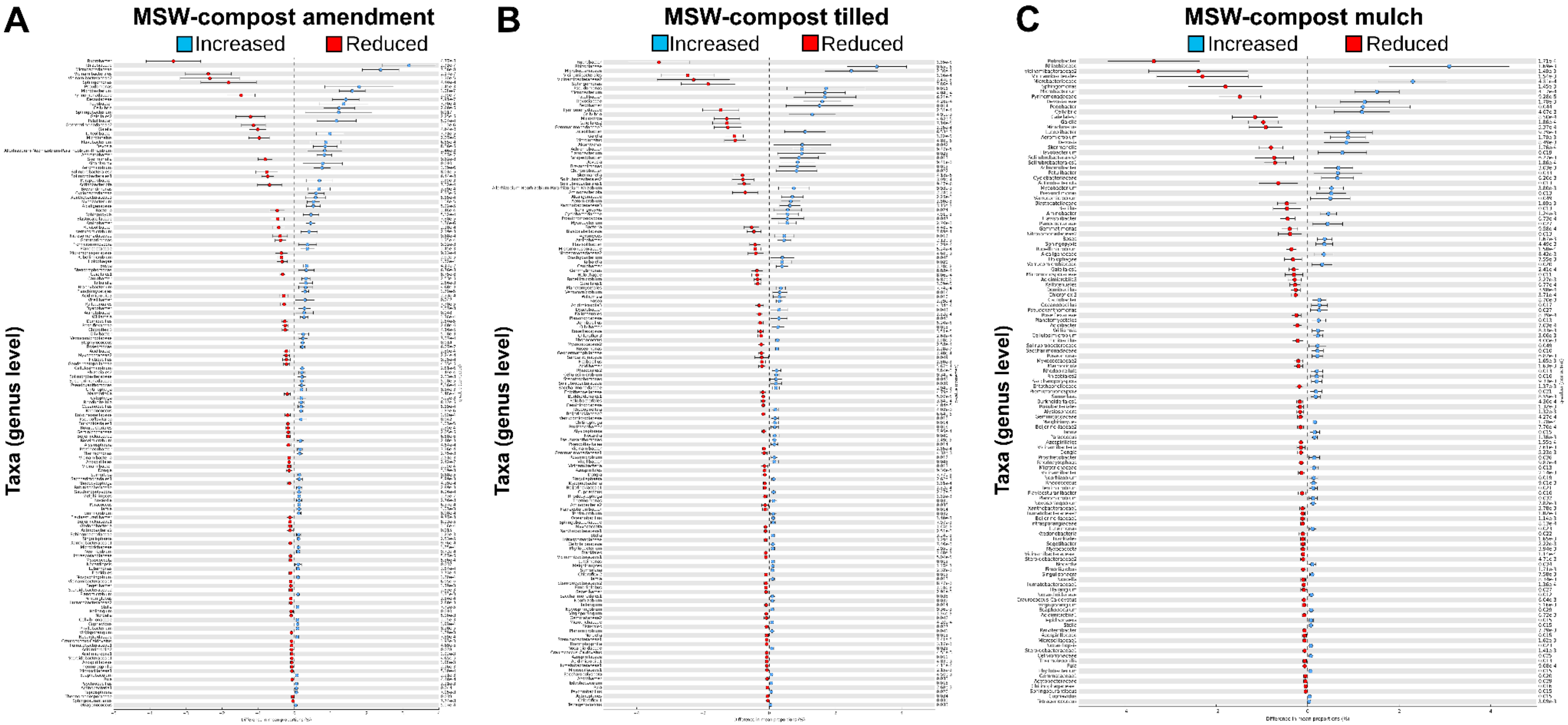

Supplementary Figure S6. STAMP Extended error bar plot, showing the response of bacterial taxa (genus-level) to MSW-compost addition. A) Compost *vs.* No compost samples comparison; here, Compost-mulch and Compost-tilled samples were merged and data from all sampling times were averaged. B) Compost tilled *vs.* No compost samples comparison. C) Compost mulch *vs.* No compost samples comparison. The comparison was performed with Welch's test. Genera showing both fold change ≥2 and Benjamini-Hochberg-corrected *p*≤0.05 were considered as significant. Error bars indicate 95% confidence interval. Only significantly different taxa were plotted. The complete list of genus-level taxa is provided in Supplementary material (files "STAMP.genera.compost_amendment.xlsx", "STAMP.genera.compost_tilled.xlsx", and "STAMP.genera.compost_mulch.xlsx").

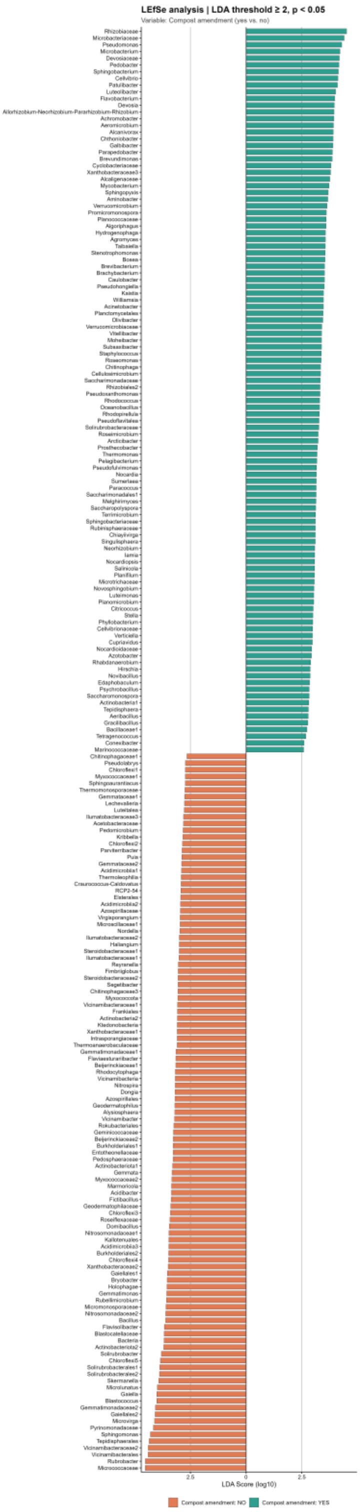

Figure S7 – LEfSe plot showing the response of bacterial taxa (genus-level) to MSW-compost addition (Compost *vs.* No compost samples comparison; Compost-mulch and Compost-tilled samples merged and data from all sampling times were averaged). Genera showing both Linear Discriminant Analysis Effect Size ≥2 and Benjamini-Hochberg-corrected $p<0.05$ were considered as significant. Only significantly different taxa were plotted. The list of these significant genus-level taxa is provided in Supplementary material (files "LEfSe.genera.compost_amendment.xlsx").

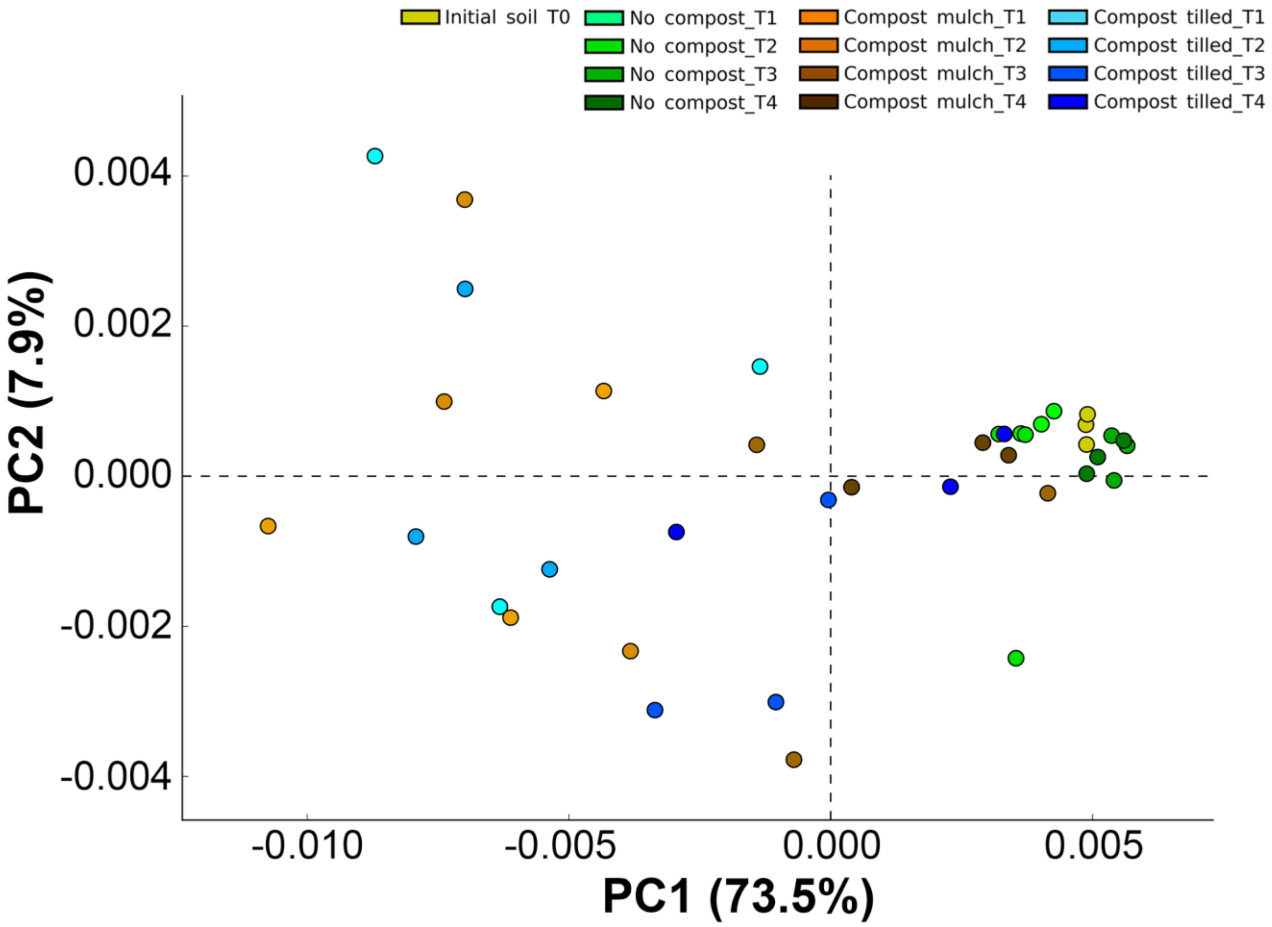

Supplementary Figure S8. STAMP PCA plot of Picrust2's predicted functional pathways, showing the relative distances between samples, colored according to treatment (base colors) and sampling time (color tones). Compost used samples were removed due to their extreme distance which would flatten all other samples.

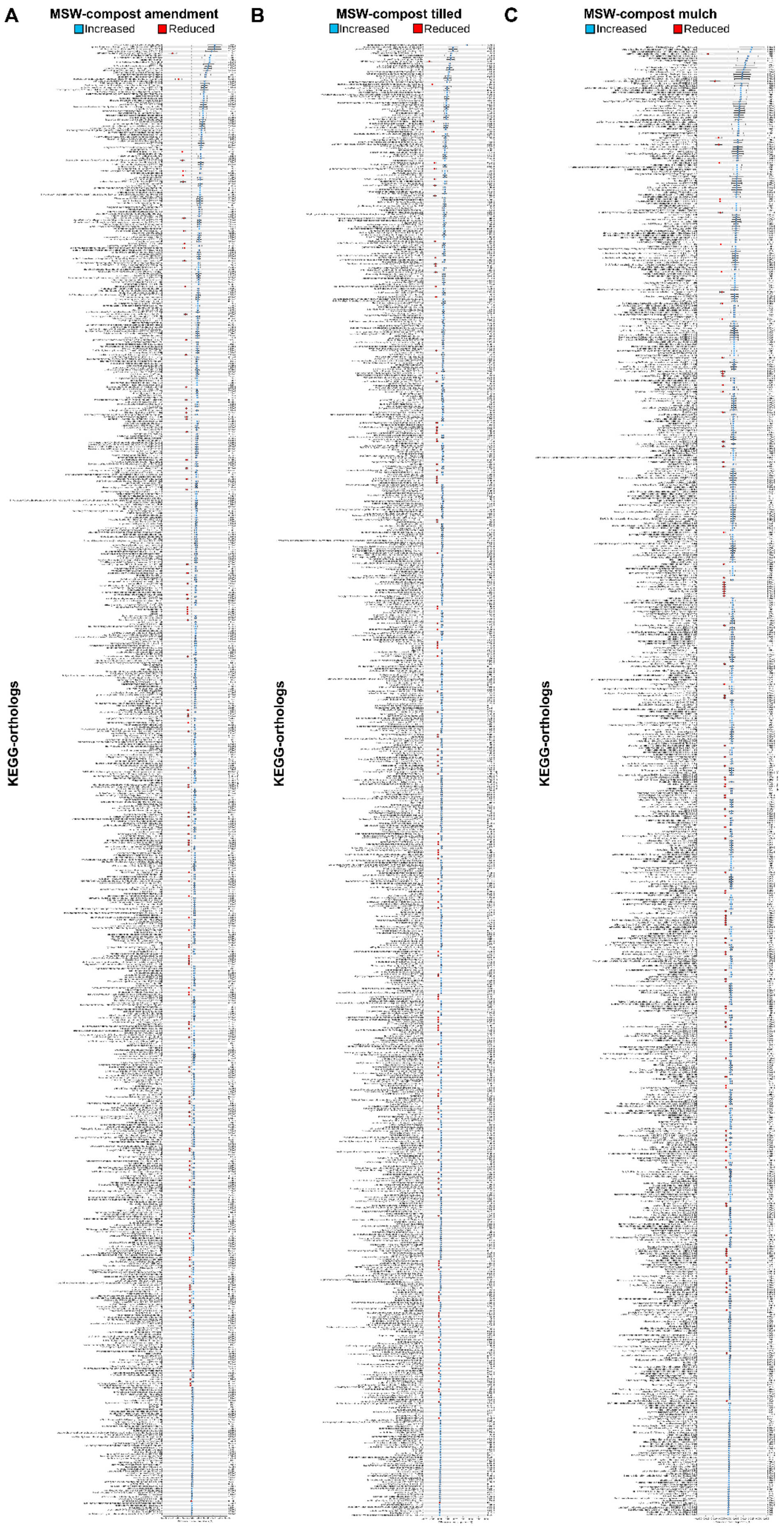
A
MSW-compost amendment
Increased
Reduced
B
MSW-compost tilled
Increased
Reduced
C
MSW-compost mulch
Increased
Reduced
KEGG-orthologs
KEGG-orthologs
KEGG-orthologs

Supplementary Figure S9. STAMP Extended error bar plots showing the response of Picrust2's predicted KEGG Orthologs to MSW-compost addition. A) Compost *vs.* No compost comparison; here, Compost-mulch and Compost-tilled samples were merged and data from all sampling times were averaged. B) Compost tilled *vs.* No compost samples comparison. C) Compost mulch *vs.* No compost samples comparison. The comparison was performed with Welch's test. Features having minimum fold change ≥2 and Benjamini-Hochberg-corrected $p \leq 0.05$ were considered as significantly affected. Error bars indicate 95% confidence interval. Only significantly different KEGG orthologs are plotted. The complete list of genus-level taxa is provided in Supplementary material (files "STAMP.Picrust2_KO.compost_amendment.xlsx", "STAMP.Picrust2_KO.compost_tilled.xlsx" and "STAMP.Picrust2_KO.compost_mulch.xlsx").

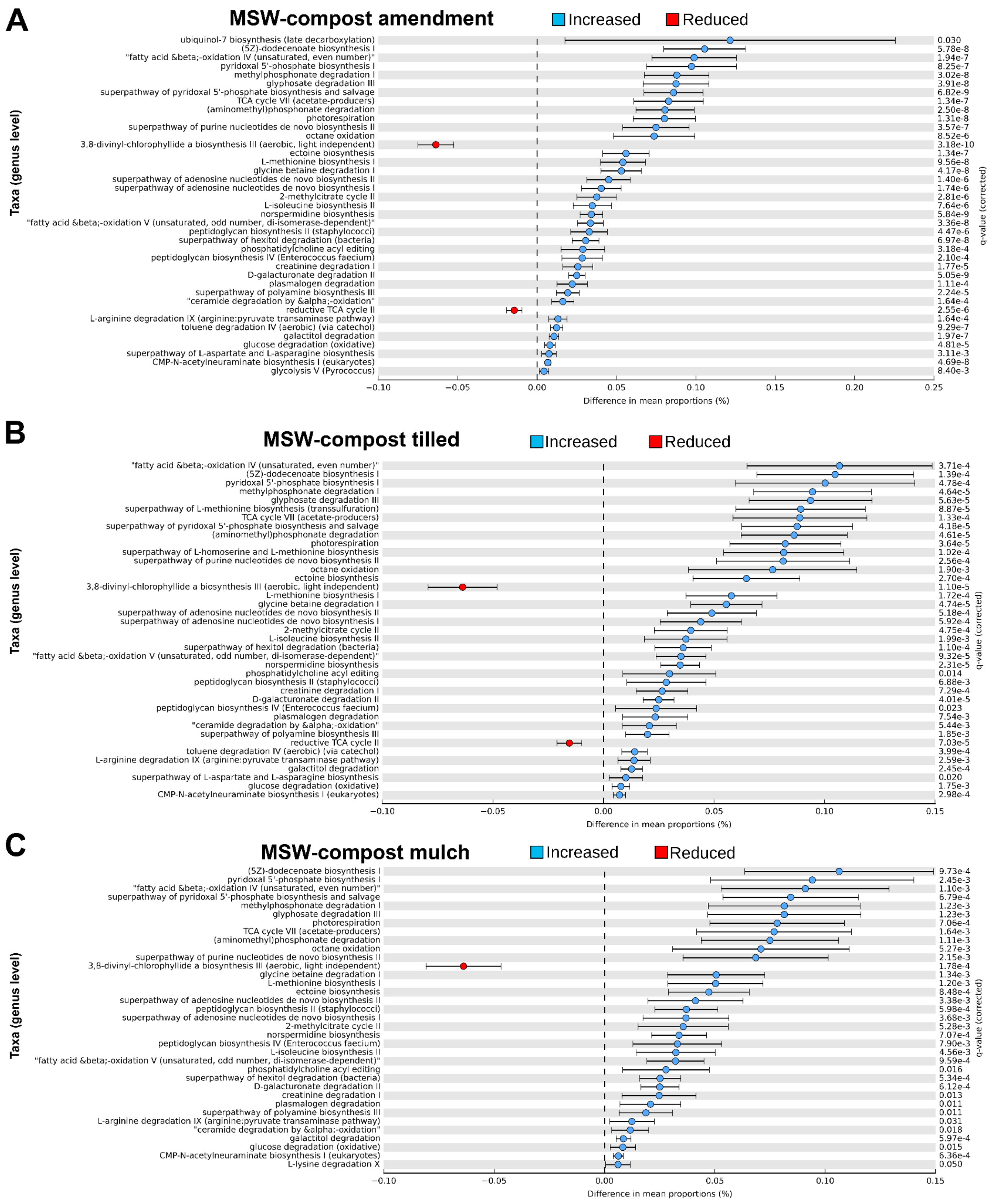

Supplementary Figure S10. STAMP Extended error bar plots showing the response of Picrust2's predicted Functional pathways to MSW-compost addition. A) Compost *vs.* No compost comparison; here, Compost-mulch and Compost-tilled samples were merged and data from all sampling times were averaged. B) Compost tilled *vs.* No compost samples comparison. C) Compost mulch *vs.* No compost samples comparison. The comparison was performed with Welch's test.

Features having minimum fold change ≥2 and Benjamini-Hochberg-corrected *p*≤0.05 were considered as significantly affected. Error bars indicate 95% confidence interval. Only significantly different Functional pathways are plotted. The complete list of genus-level taxa is provided in Supplementary material (files “STAMP.Picrust2_pathways.compost_amendment.xlsx”, “STAMP.Picrust2_pathways.compost_tilled.xlsx” and “STAMP.Picrust2_pathways.compost_mulch.xlsx”).
